# A semiautomatic tool for prostate segmentation in radiotherapy treatment planning

**DOI:** 10.1186/1471-2342-14-4

**Published:** 2014-01-25

**Authors:** Jörn Schulz, Stein Olav Skrøvseth, Veronika Kristine Tømmerås, Kirsten Marienhagen, Fred Godtliebsen

**Affiliations:** 1 Department of Mathematics and Statistics, University of Tromsø, 9037 Tromsø, Norway; 2 Norwegian Centre for Integrated Care and Telemedicine, University Hospital of North Norway, 9038 Tromsø, Norway; 3 Department of Oncology, University Hospital of North Norway, 9038 Tromsø, Norway

**Keywords:** Delineation, Ellipse model, Empirical Bayes, Prostate, Radiotherapy treatment planning, Statistical shape analysis

## Abstract

**Background:**

Delineation of the target volume is a time-consuming task in radiotherapy treatment planning, yet essential for a successful treatment of cancers such as prostate cancer. To facilitate the delineation procedure, the paper proposes an intuitive approach for 3D modeling of the prostate by slice-wise best fitting ellipses.

**Methods:**

The proposed estimate is initialized by the definition of a few control points in a new patient. The method is not restricted to particular image modalities but assumes a smooth shape with elliptic cross sections of the object. A training data set of 23 patients was used to calculate a prior shape model. The mean shape model was evaluated based on the manual contour of 10 test patients. The patient records of training and test data are based on axial T1-weighted 3D fast-field echo (FFE) sequences. The manual contours were considered as the reference model. Volume overlap (Vo), accuracy (Ac) (both ratio, range 0-1, optimal value 1) and Hausdorff distance (HD) (mm, optimal value 0) were calculated as evaluation parameters.

**Results:**

The median and median absolute deviation (MAD) between manual delineation and deformed mean best fitting ellipses (MBFE) was Vo (0.9 ± 0.02), Ac (0.81 ± 0.03) and HD (4.05 ± 1.3)mm and between manual delineation and best fitting ellipses (BFE) was Vo (0.96 ± 0.01), Ac (0.92 ± 0.01) and HD (1.6 ± 0.27)mm. Additional results show a moderate improvement of the MBFE results after Monte Carlo Markov Chain (MCMC) method.

**Conclusions:**

The results emphasize the potential of the proposed method of modeling the prostate by best fitting ellipses. It shows the robustness and reproducibility of the model. A small sample test on 8 patients suggest possible time saving using the model.

## Background

Prostate cancer is the second most diagnosed cancer accounting for 14 percent of all cancers diagnosed worldwide [1]. It is most common in males over the age of 50, and has the highest incidence rate in the developed countries. Aggressive tumors are usually treated with extern radiotherapy or brachytherapy which requires a precise treatment plan for the target volume. In any type of radiotherapy treatment, radiation of healthy tissue should be minimized while maintaining the desired dose to the target volume. Therefore, a successful treatment of prostate cancer relies on an accurate segmentation of the prostate from the surrounding tissue, by image-based description of the shape and location of the target volume. The volume of interest is characterized by a smooth shape, and for this reason an algorithmic description of the volume is feasible.

Transrectal ultrasound (TRUS), magnetic resonance (MR) and computed tomography (CT) images are the three main imaging techniques used in diagnosis, treatment planning and follow-up examination of prostate cancer. Smith et al. [2] investigated the effects of these imaging techniques on the properties of the prostate volume. A collection of methods available for prostate segmentation is reviewed by Ghose et al. [3]. In addition to the methods presented by Ghose et al., alternative approaches are available in the literature, such as the medial or skeleton representation of the prostate [4-8]. The present work proposes a segmentation method which falls into the category of deformable meshes in Ghose et al. [3], but refers to the term geometrical parametrization as described in Dryden and Mardia [9]. The main focus of this paper is the development of a statistical shape model for the prostate. An overview about this type of models in 3D medical image segmentation is presented for example by Davies et al. [10] and Heimann and Meinzer [11].

The works of Saroul et al. [12] and Mahdavi et al. [13] are related to the stacked ellipses parametrization method used in this paper. Mahdavi et al. [13] proposes a 3D ellipsoid shape of the prostate in warped transrectal 3D ultrasound images based on control points. This method extends the warping idea proposed in Badiei et al. [14] from 2D to 3D ultrasound images. On the contrary, we focus on slice-wise best fitting ellipses which will introduce more flexibility into the model, e.g., between the positions and lengths of the first and second axes of the ellipses between neighbor slices. The approach of slice-wise best fitting ellipses has similarities to a tubular medial representation [15].

Beside the single segmentation of the prostate, several attempts have been tried out for a joint segmentation of neighbor organ and structure to gain improved segmentation results [16-18].

**Figure 1:**
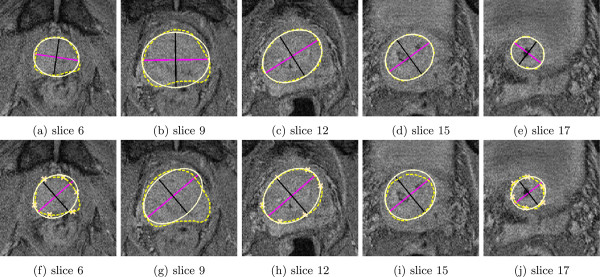
**Selected slices of MR data set 3 from the test data set. (a)-(e)** Manual delineation of the prostate (dashed line) and best fitting ellipse (solid line). **(f)-(j)** Manual delineation of the prostate (dashed line), deformed mean shape (solid line) and defined control points in the first, center and last slice.

To our knowledge, despite the substantial effort in this area, no widely implemented algorithm exists. In oncology departments this means that the physician has to delineate the prostate slice by slice. This is time-consuming and inefficient. We propose a less ambitious approach compared to more sophisticated models, such as skeletal models as discussed above, in that we use a method that gives a useful starting point for the physician after the definition of few control points. Given the initial estimate of the volume of interest, the physician can adjust the estimate according to their evaluation of the image rather than starting from scratch. By this approach, we obtain the same accuracy with less effort. The main points in our approach are as follows: First, we accept that the algorithm cannot give a fully precise description of the volume. Our main aim is therefore to give a good estimate which can be used as a starting point for the physician. Second, we use a simple ellipse model that is easy to interpret and understand. Our hypothesis is that a more efficient use of physicians in Radiotherapy Treatment Planning (RTP) of patients with prostate cancer can be obtained by an easy-to-interpret semiautomatic tool.Figure 1 shows an example of the initial estimate we typically obtain for a single image slice. The dashed line in (a) to (e) describes the manual contour while the solid line shows the best fitting ellipse including the two principal axes for the observed data of this slice. Note that the fitted model is very much in agreement with the manual line, indicating that the stacked ellipses model gives a good description of the object of interest. The solid lines in (f)-(j) shows the outcome from our model in this situation together with few defined control points.

This result shows a typical performance of the method, and that the estimate is close to the best fit we can obtain with the ellipse model. The full processing demands little computational resources, such that the suggested delineation can be presented immediately. The example is discussed further in the Methods and Results and discussion section.

The rest of the paper is organized as follows. In the Methods section, we introduce the data sources and the proposed stacked ellipses model, and discuss the shape space and statistics along with constraints and parameters. Results are presented in the Results and discussion section using a test data set to show the potential of the mean shape model, followed by a Conclusions section. Additional file 1 with further detailed discussion is available online.

## Methods

### Preliminaries

Each prostate must be described by a shape model in order to calculate statistics, e.g., by stacked ellipses as a parametric shape model. The parameters of a parametric shape model can be estimated from a training set. The training set models also the geometric variability of anatomical structures by a shape probability distribution. The training set contains volume and contour information of segmented prostates from *N* patients. The volume information describes the image modalities (e.g., CT or MR) and the contour information the volume of interest as defined in the following.

The volume information of each training set *n *= 1, …,*N* is defined by a 3-dimensional matrix *V*_*n *_where *V*_*n*_(*i*,*h*) contains the observed gray level in voxel (*i*,*h*), *i *= (*i*_1_,*i*_2_) ∈ {1,…,*I*_1_} × {1,…,*I*_2_} are the pixel indices in a slice, where typically *I*_1 _= *I*_2_, and *h *∈ {1,…,*H*} is the number of slices per data set. The number of slices *H* is not necessarily the same for all patients in the training data sets. Therefore, we indicate *H* by *H*_*n *_and in the same manner *I*_1 _by *I*_*n*1 _and *I*_2 _by *I*_*n*2_, but for simplicity we use *H*, *I*_1 _and *I*_2 _if the meaning is clear.

In addition to the volume information, each training set *n *=1,…,*N* consists of contour information of the prostate, manually drawn by a physician. The contour information can be modeled by a (*M* × *K*_*n*_) configuration matrix $X_{n}:=(X_{n1},\ldots ,X_{{\text{nK}}_{n}})$ with $X_{\text{nk}}={\left(x_{1k}^{n},x_{2k}^{n},x_{3k}^{n}\right)}^{T}\in R^{3},k=1,\ldots ,K_{n}$, where *K*_*n *_defines the total number of available contour information points in a data set and *M *= 3 defines the dimension. We assume the contour information for an object is defined in a sequentially sorted number *L*_*n *_of equidistant slices whereas each contour slice contains ${\tilde{K}}_{\text{nl}}$ contour points, *l *= 1,…,*L*_*n*_. Hence it follows $K_{n}=\underset{l}{\sum }{\tilde{K}}_{\text{nl}}$ and $X_{n}=({\tilde{X}}_{n1},\ldots ,{\tilde{X}}_{{\text{nL}}_{n}})$. The image information in slice *l* is denoted by *S*_*nl *_and $S_{n}=\{S_{n1},\ldots ,S_{{\text{nL}}_{n}}\}\subseteq V_{n}$ and ${\tilde{X}}_{\text{nl}}$ defines the configuration matrix in slice *S*_*nl*_.

In summary, the training population is given by the set {V,X}, with a set of volume information $V=\{V_{1},\ldots ,V_{N}\}$ and configuration matrices $X=\{X_{1},\ldots ,X_{N}\}$. We assume *X*_*n *_defines the configuration matrix for the corresponding data set *V*_*n *_and matches the volume information *V*_*n *_exactly.

**Figure 2:**
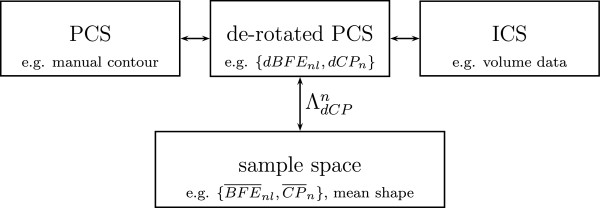
**Visualization of different coordinate systems with example data. ***PCS:* Patient based coordinate system (manual delineation line). *ICS:* Image coordinate system (volume data). *de-rotated PCS: * de-rotated patient based coordinate system with same scale and origin as PCS but same orientation as ICS (de-rotated best fitting ellipse *dBFE*_*nl*_, de-rotated control points *dCP*_*n*_). *sample space:* The transformation matrix *ΛdCPn* maps the de-rotated data {*dBFE*_*n*_,*dCP*_*n*_} to $\left\{{\bar{\text{BFE}}}_{n},{\bar{\text{CP}}}_{n}\right\}$ in the sample space.

The contour information is often defined in a Patient based Coordinate System (PCS) whereas the volume information is defined in an Image based Coordinate System (ICS). The ICS can be transformed to PCS by a transformation matrix *Λ*_*DCM*_, which transform an image coordinate *p*^*im *^= (*i*_1_,*i*_2_,*h*)^*T*^ to patient coordinate *p*^*p *^= (*x*,*y*,*z*)^*T*^. The definition of *Λ*_*DCM *_and the relation between PCS and ICS (see Figure 2) is discussed in detail in the Additional file 1. In addition, we introduce a de-rotated PCS where volume and contour information are aligned to each other.

### Modeling

**Figure 3:**
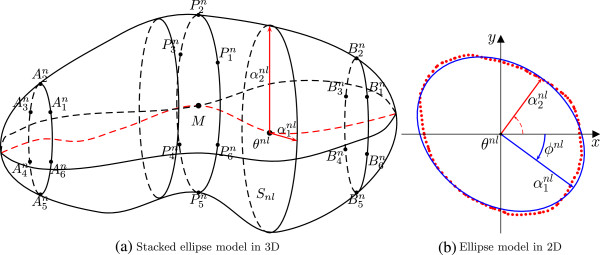
The prostate model of stacked slicewise best-fitting ellipses illustrated by (a) a 3D view of the model and (b) the corresponding 2D model of the prostate contour in a slice.

The prior information inferred from the training set is incorporated into a shape model. We assume a stacked ellipse model as a shape prior for the prostate. Specifically, the prostate outline in slice *S*_*nl*_,*l *= 1,…,*L*_*n*_, *n *= 1,…,*N* is modeled by a slicewise best-fitting ellipse, as visualized in Figure 3. An ellipse in slice *S*_*nl *_can be uniquely described by $\rho_{\text{nl}}={\left(\theta^{\text{nl}},\alpha^{\text{nl}},\phi^{\text{nl}}\right)}^{T}\in R^{2}\times R_{+}^{2}\times (-\frac{\pi }{2},\frac{\pi }{2}]$ with 

• **position**$\theta^{\text{nl}}={\left(\theta_{1}^{\text{nl}},\theta_{2}^{\text{nl}}\right)}^{T}\in R^{2}$ defines the center in slice *S*_*nl*_,

• **length of principle axes**$\alpha^{\text{nl}}={\left(\alpha_{1}^{\text{nl}},\alpha_{2}^{\text{nl}}\right)}^{T}\in R_{+}^{2}$ and

• **rotation angle**$\phi^{\text{nl}}\in (-\frac{\pi }{2},\frac{\pi }{2}]$ in slice *S*_*nl*_.

The rotation parameter *ϕ*^*nl *^is defined corresponding to the ICS with origin *θ*^*nl *^in slice *S*_*nl*_. The boundary of an ellipse *ρ*_*nl *_centered at $\theta^{\text{nl}}\in R^{2}$ in slice *S*_*nl *_is defined by

(1) $$C(\rho_{\text{nl}})=\left\{\text{Rx}+\theta^{\text{nl}}:\frac{x_{1}^{2}}{{\left(\alpha_{1}^{\text{nl}}\right)}^{2}}+\frac{x_{2}^{2}}{{\left(\alpha_{2}^{\text{nl}}\right)}^{2}}=1,x\in R^{2}\right\}$$

$$\text{and}\;R=\left(\begin{array}{cc} \text{cos}\phi^{\text{nl}} & -\text{sin}\phi^{\text{nl}}\\ \text{sin}\phi^{\text{nl}} & \;\;\text{cos}\phi^{\text{nl}} \end{array}\right)$$

is a rotation matrix in $R^{2}$ with rotation angle *ϕ*^*nl *^and *x *= (*x*_1_,*x*_2_)^*T*^.

The shape model described in this section requires the best fit of an ellipse *C *(*ρ*_*nl*_) to the contour information ${\tilde{X}}_{\text{nl}}$ in each slice, i.e., we model ${\tilde{X}}_{\text{nl}}=C(\rho_{\text{nl}})+\varepsilon$ where *ε* is an error with mean zero. The best-fitting ellipses provide us with a slice-by-slice parametrization of the prostate for all slices in each training shape.

The problem of fitting an ellipse to geometric features like the contour is discussed widely in the literature (e.g., [19,20]). This work follows Ahn et al. [19], who proposed a least-square minimizer for ${\tilde{X}}_{\text{nl}}$. The nonlinear estimate of parameters $\rho_{\text{nl}}={\left(\theta_{1}^{\text{nl}},\theta_{2}^{\text{nl}},\alpha_{1}^{\text{nl}},\alpha_{2}^{\text{nl}},\phi^{\text{nl}}\right)}^{T}$ given ${\tilde{X}}_{\text{nl}}$ must minimize the error

$$g({\hat{\rho }}_{\text{nl}})={\left({\tilde{X}}_{\text{nl}}-\tilde{C}({\hat{\rho }}_{\text{nl}})\right)}^{T}\left({\tilde{X}}_{\text{nl}}-\tilde{C}({\hat{\rho }}_{\text{nl}})\right)$$

where $\tilde{C}({\hat{\rho }}_{\text{nl}})$ is a set of nearest orthogonal points of ${\tilde{X}}_{\text{nl}}$ to $C({\hat{\rho }}_{\text{nl}})$.

#### 

**Definition 1** (Best fitting ellipse (BFE)). A best fitting ellipse for slice *S*_*nl *_is defined by the set ${\text{BFE}}_{\text{nl}}:={\left(\theta^{\text{nl}},\alpha^{\text{nl}},\phi^{\text{nl}}\right)}^{T}\in R^{2}\times R_{+}^{2}\times (-\frac{\pi }{2},\frac{\pi }{2}]$, *l *= 1,…,*L*_*n*_, *n *= 1,…,*N *and minimizes the error function *g*, i.e., ${\text{BFE}}_{\text{nl}}={\hat{\rho }}_{\text{nl}}$ with

(2) $$g({\hat{\rho }}_{\text{nl}})=\underset{\rho_{\text{nl}}\in R^{2}\times R_{+}^{2}\times (-\frac{\pi }{2},\frac{\pi }{2}]}{\text{min}}g(\rho_{\text{nl}}).$$

The first and second principal axes must be reordered after calculation of ${\text{BFE}}_{n}=\{{\text{BFE}}_{n1},\ldots ,{\text{BFE}}_{{\text{nL}}_{n}}\}$ in order to establish correspondence between parameters of adjacent slices and across the population. Improved correspondence will support accurate statistics. The basic idea in our reordering procedure is to carry out the reordering corresponding to the lowest rotation angle of both principal axes to the first principal axis of the neighbor slice where the center slice is chosen as the basis. The rotation between the center slice *M* and an arbitrary slice is constrained by max(|*ϕ*^*i *^- *ϕ*^*M*^|) = *π*,* i*∈ {1,…,*L*} after reordering. Therefore, the set *BFE*_*n *_of reordered best-fitting ellipses is an element of $(R^{2}\times R_{+}^{2}\times {\left(-\pi ,\pi ]\right)}^{L_{n}}$.

A further improvement of correspondence is achieved by the introduction of two additional constraints in the parameter model.

First, we relax the rotation parameter *ϕ*^*nl *^in case of circularity. If both principal axes have the same length, the orientation of an ellipse is undefined. Therefore we penalize *ϕ*^*nl *^in the case of high circularity by taking $\phi^{nl^{\prime }}$ from the neighboring slices into account. Second, smoothing is performed between neighboring slices to avoid large forward and backwards rotations between *ϕ*^*n *(*l *- 1)^,*ϕ*^*nl *^and *ϕ*^*n *(*l *+ 1)^. The reordering algorithm and implementation of constraints are described in detail in Additional file 1.

The current implementation assumes the definition of control points *CP*_*n *_in the training data set {*V*_*n*_,*X*_*n*_,*BFE*_*n*_}, where ${\text{BFE}}_{n}\in (R^{2}\times R_{+}^{2}\times {\left(-\pi ,\pi ]\right)}^{L_{n}}$ is a reordered set of best fitting ellipses, *n *= 1,...,*N*. Furthermore, the control points have to be defined manually by a physician in a new patient data set. The control points are used to make the best fitting ellipses *BFE*_*n *_comparable and to transform the parametrized ellipses model to a common position, scale and orientation by a transformation matrix *ΛdCPn*. The transformation matrix *ΛdCPn *maps the de-rotated prior data {*dBFE*_*n*_,*dCP*_*n*_} to $\left\{{\bar{\text{BFE}}}_{n},{\bar{\text{CP}}}_{n}\right\}$ in the sample space, as depicted in Figure 2. In this article, we assume 6 control points in the first, center and last slice at the boundary of the prostate, i.e.,

$${\text{CP}}_{n}=\left\{A_{1}^{n},\ldots ,A_{6}^{n},P_{1}^{n},\ldots ,P_{6}^{n},B_{1}^{n},\ldots ,B_{6}^{n}\right\}$$

as visualized in Figure 3. In addition, we have tested alternative control point configurations. They are described together with the construction of *ΛdCPn *in Additional file 1.

After transformation we have obtained a reordered and comparable set of best fitting ellipses

$${\bar{\text{BFE}}}_{n}=\left\{{\bar{\text{BFE}}}_{n1},\ldots ,{\bar{\text{BFE}}}_{{\text{nL}}_{n}}\right\}$$

with ${\bar{\text{BFE}}}_{\text{nl}}={\left({\bar{\theta }}^{\text{nl}},{\bar{\alpha }}^{\text{nl}},{\bar{\phi }}^{\text{nl}}\right)}^{T}$, *n *= 1,…,*N*, *l *= 1,…,*L*_*n*_. The statistical analysis of the training data requires an equal number *L*_1 _= … = *L*_*N *_to establish correspondence between the parameters of the best fitting ellipses. Therefore, we interpolate the set ${\bar{\text{BFE}}}_{\text{nl}}$ to a common number *L*.

When *L* is chosen, interpolation is done by independent cubic interpolation in each dimension, i.e., we find points of a one-dimensional function that underlies the data ${\bar{\theta }}_{1}^{\text{nl}},{\bar{\theta }}_{2}^{\text{nl}},{\bar{\theta }}_{3}^{\text{nl}},{\bar{\alpha }}_{1}^{\text{nl}},{\bar{\alpha }}_{1}^{\text{nl}}$ and ${\bar{\phi }}^{\text{nl}}$. The final interpolated best fitting ellipses are denoted by

(3) $${\text{iBFE}}_{n}=\left\{{\text{iBFE}}_{n1},\ldots ,{\text{iBFE}}_{\text{nL}}\right\}.$$

These ellipses are used for the statistical analysis and computation of a mean shape model. To keep things simple, we denote such a reordered, transformed and interpolated set of best-fitting ellipses by *BFE*_*nl *_= (*θ*^*nl*^,*α*^*nl*^,*ϕ*^*nl*^)^*T *^for the number *L* of contour slices with *l *= 1,…,*L* and *n *= 1,…,*N*. The comparable set of best fitting ellipses *BFE*_*n *_ is an element of the shape space $(R^{2}\times R_{+}^{2}\times {\left(-\pi ,\pi ]\right)}^{L}$.

### Statistical analyses

After reconstruction of our shape space we estimate the expectation and variance of the parameters of a mean shape model *μ*_*BFE *_= {*μ BFE *1,…,*μ BFEL*} with $\mu_{\text{BFE}}^{l}=(\mu_{\theta }^{l},\mu_{\alpha }^{l},\mu_{\phi }^{l})$ from the training set *BFE*_*nl*_, *l *= 1,…,*L*. We denote the mean shape mean best fitting ellipses (MBFE). In addition to the described ellipse parameters we define the position $\theta^{\text{nl}}={\left(\theta_{1}^{\text{nl}},\theta_{2}^{\text{nl}},\theta_{3}^{\text{nl}}\right)}^{T}$ in terms of a distance vector *η*^*nl *^of *θ*^*nl *^to a center curve defined by the control points. We model *θ*^*l *^= *ξ*^*l *^+ *η*^*l*^, where *ξ*^*l *^is analytically defined by *L* intersection points of the curve within each slice. Thereby, we are describing the mean shape which is closest to the control points. This approach is reasonable under the assumption that the control points are well defined. In Additional file 1 we explore various ways of describing the position parameter for different control point methods.

The mean curve of the expected location is given by

(4) $$\mu_{\theta_{j}}^{l}=\frac{1}{N}\overset{N}{\underset{i=1}{\sum }}\theta_{j}^{\text{il}},\;j\in \left\{1,2,3\right\},$$

where $\mu_{\theta }^{l}={\left(\mu_{\theta_{1}}^{l},\mu_{\theta_{2}}^{l},\mu_{\theta_{3}}^{l}\right)}^{T}$, *l *= 1,…,*L*. The variance and covariance are estimated by

(5) $${\left(\sigma_{\theta_{j}}^{l}\right)}^{2}=\frac{1}{N-1}\overset{N}{\underset{i=1}{\sum }}{\left(\theta_{j}^{\text{il}}-\mu_{\theta_{j}}^{l}\right)}^{2},\;j\in \{1,2,3\},\text{and}$$

(6) $$\Sigma_{\theta }^{l}=\frac{1}{N-1}\overset{N}{\underset{i=1}{\sum }}(\theta^{\text{il}}-\mu_{\theta }^{l}){\left(\theta^{\text{il}}-\mu_{\theta }^{l}\right)}^{T}.$$

The length parameter is modeled by a log-normal distribution because $\alpha \in R_{+}^{2}$. Thus we estimate the mean and variance of $a=\text{log}(\alpha )\in R^{2}$. The estimation of means and variances of the remaining parameters *a*,*ϕ*,*η *is according to (4-5).

Following Dryden and Mardia [9] we suggest a prior distribution for a new data set as

$$\begin{array}{lll} & \theta_{1}^{l}∼N\left(\mu_{\theta_{1}}^{l},{\left(\sigma_{\theta_{1}}^{l}\right)}^{2}\right),\;\theta_{2}^{l}∼N\left(\mu_{\theta_{2}}^{l},{\left(\sigma_{\theta_{2}}^{l}\right)}^{2}\right),\; & \\ & a_{1}^{l}∼N\left(\mu_{a_{1}}^{l},{\left(\sigma_{a_{1}}^{l}\right)}^{2}\right)\Leftrightarrow \; & \\ & \alpha_{1}^{l}∼\text{log-}N\left(\mu_{a_{1}}^{l},{\left(\sigma_{a_{1}}^{l}\right)}^{2}\right)\text{with}a_{1}^{l}=\text{log}(\overset{l}{\underset{1}{\alpha }}),\; & \\ & a_{2}^{l}∼N\left(\mu_{a_{2}}^{l},{\left(\sigma_{a_{2}}^{l}\right)}^{2}\right)\Leftrightarrow \; & \\ & \alpha_{2}^{l}∼\text{log-}N\left(\mu_{a_{2}}^{l},{\left(\sigma_{a_{2}}^{l}\right)}^{2}\right)\text{with}a_{2}^{l}=\text{log}(\overset{l}{\underset{2}{\alpha }}),\; & \\ & \phi^{l}∼N\left(\mu_{\phi }^{l},{\left(\sigma_{\phi }^{l}\right)}^{2}\right),\; & \end{array}$$

*l *= 1,…,*L*. If *θ*^*l *^is defined according to the center curve given by the control points as described above, we model $\eta_{i}^{l}∼N\left(\mu_{\eta_{i}}^{l},{\left(\sigma_{\eta_{i}}^{l}\right)}^{2}\right),i=1,2$. Since the rotational parameter is expected to have small variance it is not necessary to apply a circular distribution, and we assume normality.

After constructing the shape model we estimate the best fitting ellipse *BFE*_*l *_parametrized by *ρ*_*l *_= (*θ*^*l*^,*α*^*l*^,*ϕ*^*l*^)^*T*^, *l *= *l*,…,*L* in a new data set given the control points *CP*. This is obtained through the posterior *π *(*ρ *∣ *S*) where *s*_*il *_∈ *S *⊆ *V* is the volume information and *i *= (*i*_1_,*i*_2_) ∈ *I *(*ρ*) is a set of indices within the ellipses *ρ*. The control points *CP * are used to deform the prior model *π *(*ρ*). Therefore we model the posterior by an empirical Bayes approach [21]. The posterior

(7) π(ρ∣S,CP)∝L(S∣ρ)∗π(ρ∣CP)

defines the posterior density of the deformed template *π *(*ρ *∣ *CP*) given the the observed image. The Likelihood or image model *L *(*S * ∣ *ρ*) is the joint probability density function of the gray levels given the parametrized object *ρ *|_*CP*_, while *ρ *|_*CP *_defines the ellipses *ρ *deformed by the control points *CP*. The prior *π *(*ρ *∣ *CP*) models realistic variations from our mean shape $\mu_{\text{BFE}}\in {\left(R^{2}\times R_{+}^{2}\times (-\pi ,\pi ]\right)}^{L}$ given the control points. We are estimating the posterior distribution using a Markov chain Monte Carlo (MCMC) approach. The method and results are discussed in detail in Additional file 1.

### Evaluation

We have evaluated the proposed method using 33 patient case studies. The training data set consists of *N *= 23 T1-weighted Fast Field Echo (FFE) 3D Magnetic Resonance (MR) data. The mean shape model and variance is calculated from the training data set and applied to a test data set of 10 MR FFE case studies. The splitting in test and training data is done according to the sequence of data acquisition. Each data set consists of *H*_*n *_Digital Imaging and Communications in Medicine (DICOM) image files and one DICOM region structure file, while the contour information of the prostate is stored in the header of a DICOM file without any image information. The voxel size (*l*_*x*_,*l*_*y*_,*l*_*z*_) is (0.559mm,0.559mm,3mm) with a slice distance of 3.3mm of the data sets *V*_*n*_. Each slice consists of 288 × 288 voxels. The average number of slices with manual prostate contour information is 10.478±2.626 (mean±standard deviation) in the training set and 10.5 ± 2.799 in the test set where the mean is given by $\mu_{L}=\frac{1}{N}\overset{N}{\underset{n=1}{\sum }}L_{n}$ and the standard deviation by ${\left(\frac{1}{N-1}\overset{N}{\underset{n=1}{\sum }}{\left(L_{n}-\mu_{L}\right)}^{2}\right)}^{\frac{1}{2}}$. Figure 1 illustrates test patient 3, whose image set consists of 24 MR FFE slices whereas 12 slices contain contour information. Slice 6 is the first slice where contour information of the prostate is available and the last slice is 17.

An ethics approval was not required for this study under Norwegian law because the aim of this study was to develop a tool and not to obtain new knowledge on medicine or diseases. The study uses solely data that were collected during routine medical treatment independent of this study at the University hospital Northern Norway (UNN). Data was provided after a full anonymization following the required guidelines at UNN.

Three metrics are used to compare the manual and the semi-automatic contours. In the axial slices, where the expert manual delineations are present, we calculate the Hausdorff distance (HD) by

(8) $$d_{\text{hd}}(X,Y)=\text{max}\left\{\underset{x\in X}{\text{max}}\underset{y\in Y}{\text{min}}d(x,y),\underset{y\in Y}{\text{max}}\underset{x\in X}{\text{min}}d(x,y)\right\}.$$

The Hausdorff distance measures the maximum distance of a point in a set *X* to the nearest point in *Y* or vice versa. Generalization to 3D uses mean or median over all slices. The measure indicates how much manual corrections are required. An ideal value of HD equal to zero reflects complete agreement of the contours. In addition, the number of slices with a HD greater than 3 mm is reported and compared to the total number of slices with prostate information. A threshold of 3 mm is often seen as clinically acceptable [22]. A second criteria is the volume overlap (or Dice similarity coefficient) defined by

(9) $$d_{\text{vol}}(X,Y)=2\frac{\left|X\cap Y\right|}{\left|X|+|Y\right|},$$

where |·| is the number of voxels contained in a region. Finally, accuracy is defined as

(10) $$d_{\text{acc}}(X,Y)=1-\frac{\left|\text{FP}|+|\text{FN}\right|}{\left|\text{TP}|+|\text{FN}\right|}$$

with *TP *= *X *∩ *Y* volume included in both *X* and *Y* (true positive), *FN *= *X *∩ (¬*Y*) volume of *X* not included in *Y* (false negative) and *FP *= (¬*X*) ∩ *Y* volume of *Y* not included by *X* (false positive). Both values range from 0 to 1, with optimal value 1. Volume overlap indicates how much of the prostate has been detected by the approach while accuracy shows how incapable the method is to select the true prostate pixels.

In addition to the quantitative metrics, we have performed a small pilot test on 8 new patients comparing time expenditure using the proposed method and manual delineation. The time expenditure for the proposed method includes the definition of control points and the correction of the contour obtained by the method for each patient. The used mean shape model and variance was calculated from the training data as described above. Time measurements were obtained by two independent physicians for each case. Manual delineations, definition of control points and corrections were performed using the treatment planning system Eclipse^TM^.

## Results and discussion

The evaluation is performed on the deformed mean best fitting ellipses, i.e., on *π* (*ρ *∣ *CP*) in formula (7). Additional evaluations are done in Additional file 1 for *π *(*ρ *∣ *S*,*CP*).

Table 1 contains the distance metrics defined in (8) - (10) comparing the manual delineation and BFE for each test data set, and comparing the manual delineation and the deformed MBFE described by *π *(*ρ *∣ *CP*). The high Dice similarity coefficient and accuracy values and small Hausdorff distances between manual delineations and BFE confirm the stacked ellipses model. This is also reflected by the count of slices with a HD greater than 3 mm in each test data set. Only 7.6*%* of the in total 105 slices have a HD greater than 3 mm. The values show the best possible description of the test cases by the proposed model. Furthermore, Table 1 presents the metrics comparing the manual delineation and the deformed MBFE for each test data set. The distance metrics reveal the fairly accurate results. The values indicate that the deformed MBFEs used as initial contours for final delineations will lower the time expenditure of the delineation procedure. However, more slices with a HD greater than 3 mm can be observed, 59% of the 105 slices. Particularly, the test sets 3, 7 and 10 have a ratio greater than 75%. Figures 1a to 1e illustrate 5 slices of the BFE evaluation of test patient 3 from Table 1. Figures 1f to 1j illustrate 5 slices of the deformed MBFE evaluation of test patient 3 from Table 1 with a volume overlap of the manual delineation line and the deformed mean shape of 0.90 and accuracy 0.81.

**Table 1:** Evaluation metrics comparing BFE to manual delineations, and comparing deformed MBFE to manual delineations

**Test set**	**1**	**2**	**3**	**4**	**5**	**6**	**7**	**8**	**9**	**10**
**BFE**										
Dice 3D	0.96	0.97	0.95	0.96	0.94	0.94	0.96	0.96	0.97	0.93
Accuracy	0.93	0.94	0.91	0.91	0.88	0.88	0.92	0.93	0.94	0.85
HD mean	1.32	1.09	2.24	1.34	1.94	1.61	1.41	1.65	1.59	2.49
*#*HD > 3mm	0 (6)	0 (8)	3 (12)	0 (8)	2 (11)	0 (13)	0 (9)	0 (15)	1 (13)	2 (10)
**Deformed MBFE**										
Dice 3D	0.92	0.93	0.90	0.91	0.88	0.84	0.92	0.88	0.89	0.88
Accuracy	0.84	0.84	0.81	0.82	0.74	0.70	0.84	0.73	0.80	0.74
HD mean	2.38	2.58	6.12	2.76	3.79	4.32	2.73	5.59	4.88	4.63
*#*HD > 3mm	1 (6)	1 (8)	9 (12)	4 (8)	7 (11)	8 (13)	2 (9)	13 (15)	8 (13)	9 (10)

(unit: Dice 3D and accuracy in percentage, HD mean in mm, number of slices with HD ≥ 3mm compared to the total number of slices in brackets).

**Figure 4:**
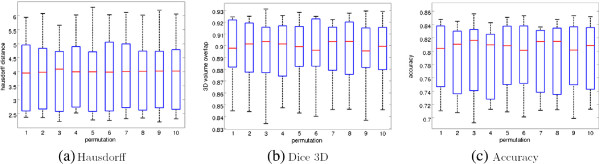
**Evaluation results between MBFE and manual delineations for 10 random permutations in training and test sets consisting of 23 and 10 patients, respectively.** The Hausdorff distance is depicted in **(a)**, the percent-volume overlap measured by the Dice coefficient in **(b)** and the accuracy in **(c)**. The central mark is the median, the edges of the box are the 25th and 75th percentiles and the whiskers extend to the extreme data points.

Table 2 shows the median and median absolute deviation (MAD) for the data groups “test data”, “training data” and “all data”. A BFE volume overlap for all data of 0.954 ± 0.010 (median ±MAD) and accuracy of 0.908 ± 0.020 confirm the model further (Table 2). Similar values in the subset of test data and training data are indicating model robustness. In addition to the BFE results, Table 2 summarizes the results by median and MAD of the distances between manual delineation and the deformed MBFE. A median volume overlap of 0.899 ± 0.021 and accuracy of 0.807 ± 0.035 of the test data show further the power of the prior. The deformation of the prior is done by the control points and can be computed directly since there is no sampling or estimation involved at this point.To evaluate the robustness of the model, we randomly split 10-times the set of 33 patients into a training set with 23 cases and a test set with 10 cases. Figure 4 shows the evaluation distances between the manual delineation and the deformed MBFE. The central mark is the median, the edges of the box are the 25th and 75th percentiles and the whiskers extend to the extreme data points. The figure shows only small variation between the different permutations, thereby demonstrating robustness of the stacked ellipses model.

**Table 2:** Evaluation metrics comparing BFE/MBFE and manual delineations for different data groups

**Data group**		**Test data**		**Training data**		**All data**	
		**BFE**	**MBFE**	**BFE**	**MBFE**	**BFE**	**MBFE**
HD mean [mm]	median	1.604	4.052	1.840	3.806	1.810	3.806
	MAD	0.274	1.305	0.286	0.427	0.289	0.538
Dice 3D [pct]	median	0.959	0.899	0.954	0.903	0.954	0.903
	MAD	0.008	0.021	0.007	0.013	0.010	0.019
Accuracy [pct]	median	0.918	0.807	0.908	0.800	0.908	0.800
	MAD	0.015	0.035	0.014	0.031	0.020	0.036

Results from using MCMC to further optimize the delineation, as described in the Methods section, are only presented in Additional file 1 since a slight improvement comes at the cost of large computation time.

The time comparisons indicated an average of 30% time saving using the proposed method compared to manual delineation. The time measurement of the proposed method includes the definition of controls points as well as the correction of the estimated contour by the physician.

## Conclusions

The presented results demonstrate the potential of the proposed method in modeling the prostate by slicewise best fitting ellipses. Deformation of the mean shape using control points gives very good results with little computational cost. Hence we believe that providing physicians with a good initial contour is beneficial in the clinical praxis of radiotherapy treatment.

The corrections of generated delineations based on few control points were not streamlined in the workflow of the physicians, and the task of correcting contours is not part of their everyday activity. Furthermore, corrections were not done directly after the definition of the control points and sometimes by different physicians, and physicians had to deal with a different orientation of the data set in the treatment planning system than in the diagnostic MRI. These issues must and can be solved for a well designed system. Therefore, a time saving of 30% likely represent a lower limit, and has to be validated in a well designed and properly powered study. Furthermore, we expect larger time savings in data sets where the prostate is imaged in a higher number of slices. In the extreme case, if the prostate is visible in only three slices, the BFE approach would not give any benefit using the current control point method. The study of inter/intra-observer variability using the proposed method compared to manual delineation was considered to be beyond the scope of this study and is left open for interesting future work.

In addition, the results show a precise description of the prostate by the BFE model with an average volume overlap of 95%. The high performance of the deformed mean shape model using the control points explains the small improvement by applying MCMC. Nevertheless, an improvement of the likelihood in the posterior distribution or by an active appearance model [23] is a field of further research as elaborated in Additional file1. A clear disadvantage of an additional method like MCMC is the extra computation time.

Further improvements can be achieved in the constraint and regularization terms, e.g., by considering the surface curvature versus changes of the ellipses parameters. We do not expect abrupt changes between neighboring slices around the central slice, but larger changes between slices towards the ends can be permitted, particularly in the length of the first and second principal axis. Also, the reduction of manual interaction in the proposed method is left for future work.

## Competing interests

The authors declare that they have no competing interests.

## Authors’ contributions

JS conducted the design, development of the methodology and the implementation of the study resulting in a stacked ellipsoid model generation from a training data set and the registration of the model in a new patient data set. Furthermore, JS drafted and revised the manuscript. SOS and FG contributed to the conception and design of the study, and helped to draft the manuscript. VKT and KM coordinated and carried out the acquisition of patient data. All authors read and approved the final manuscript.

## Pre-publication history

The pre-publication history for this paper can be accessed here:

http://www.biomedcentral.com/1471-2342/14/4/prepub

## Supplementary Material

Additional file 1 **Supplementary materials.** Article containing *i.)* a detailed description of the relative coordinates systems (e.g. ICS, PCS) on the basis of the DICOM file structure, *ii.)* post-processing procedures as for example reordering and introduction of constraints, *iii.)* a discussion of different control point method with construction of the transformation matrix $\Lambda_{\text{dCP}}^{n}$ and the parameter *η*^*nl*^, *iv.)* elaboration of the posterior distribution, and *v.)* a section with additional data analysis. Click here for file
