# Supplementary material for: A semiautomatic tool for prostate segmentation in radiotherapy treatment planning
Source: BMC Med Imaging. 2014 Jan 25;14:4. doi: 10.1186/1471-2342-14-4 (PMC3933010; doi:10.1186/1471-2342-14-4)
Supplement: Additional file 1 — Supplementary materials. Article containing i.) a detailed description of the relative coordinates systems (e.g. ICS, PCS) on the basis of the DICOM file structure, ii.) post-processing procedures as for example reordering and introduction of constraints, iii.) a discussion of different control point method with construction of the transformation matrix ΛdCPn and the parameter η nl , iv.) elaboration of the posterior distribution, and v.) a section with additional data analysis. [file 1471-2342-14-4-S1.pdf]

Supplementary material:
A semiautomatic tool for prostate segmentation in radiotherapy treatment planning

Jörn Schulz*[1], Stein Olav Skrøvseth[2] and Fred Godtliebsen[1]

[1]Department of Mathematics and Statistics, University of Tromsø, 9037 Tromsø, Norway
[2]Norwegian Centre for Integrated Care and Telemedicine, University Hospital of North Norway, 9038 Tromsø, Norway

Email: Jörn Schulz*- jorn.schulz@uit.no; Stein Olav Skrøvseth - stein.olav.skrovseth@telemed.no; Fred Godtliebsen - fred.godtliebsen@uit.no;

*Corresponding author

1 Data and relative coordinate systems

In this section, the volume data, particular on the basis of the DICOM data structure and the corresponding relative coordinate systems are discussed.

1.1 Basic principles

Rotations in the three dimensional space can be represented by the axis–angle representation [1] where an axis $c \in S^2$ is a direction that is left fixed by the rotation and angle θ is the amount of rotation. The pair (c, θ) represents a rotation in 3-space. As a convention, the orientation of the angle is determined by the right-hand rule, and a vector is a column vector. A vector $v \in \mathbb{R}^3$ rotated by (c, θ) can be obtained by pre-multiplication of the corresponding rotation matrix

$$R(c, \theta) = I_3 + \sin\theta [c]_\times + (1 - \cos\theta)(cc' - I_3), \tag{1}$$

where $[c]_\times$ is the cross product matrix, so that $[c]_\times x = c \times x$ for any $x \in \mathbb{R}^3$.

Definition 1 (Extended map)**.** The map ext maps a vector $v \in \mathbb{R}^n$ from a Cartesian coordinate system to a vector $(v, \tau)^T \in \mathbb{R}^n \times \{0, 1\}$ in an extended coordinate system. Furthermore we denote with ext^{-1} the related inverse map.

1.2 Volume information

We obtain from Magnetic Resonance (MR) tomography a discrete volume information of a continuous part of the lower male abdomen which contains the prostate. The discrete volume information of a patient data set is given by $I_1 \times I_2 \times H$ voxels on a regular grid in three dimensional space. We describe a voxel by a cube with middle point (x, y, z) and length l_x, width l_y and height l_z. Other approaches are possible too. An easy model is to assume the value of a voxel is given by the mean intensity about the volume with an overlap in x-y-direction and small gaps in z-direction, i.e., we assume

$$V(i,h) = \mu + \varepsilon_{ih} \text{ where } \varepsilon_{ih} \sim N(0, \sigma^2) \text{ iid}$$

and $i = (i_1, i_2) \in \{1, \ldots, I_1\} \times \{1, \ldots, I_2\}$, $h \in \{1, \ldots, H\}$. A more complex approach could up-rate the central areas of a voxel. The value of a voxel (i.e. the mean intensity) may describe different properties.

1.3 DICOM files and coordinate system

The Digital Imaging and Communications in Medicine (DICOM) file is a common standard container file used particularly in medical image processing. The file structure consists of a header part containing general data information and an image information part which contains mean intensities of the voxels. DICOM files uses a right handed Left-Posterior-Head (LPH) coordinate system. In a right handed coordinate system the x-axis is defined by the the thumb, the y-axis by the first finger and the z-axis by the second finger of the right hand. The coordinate system is related to the patient and not to the scanner. In a DICOM data set usually one file is defined per slice. This is the case in our data sets too. In terms of image processing, the most important variables in each file are

Rows (scalar) defines the number I_1 of voxels in row direction.

Columns (scalar) defines the number I_2 of voxels in column direction.

ImagePositionPatient (IPP) (3×1 vector) defines the coordinate of center of top left voxel in a slice.

ImageOrientationPatient (IOP) (6×1 vector) defines the unit vector along an image row and the unit vector along a column.

PixelSpacing (2×1 vector) which determine at first row spacing then column spacing, i.e. $PixelSpacing = (l_x, l_y)^T$.

These five variables define uniquely a slice in $\mathbb{R}^3$. Figure 1 visualizes some key parameters for a slice. The two variables PixelSpacing and IPP are defined in millimeter whereas IOP defines two unit vectors. Note, the variable SliceThickness defined in the DICOM file header may not be equal to the slice distance between two neighbor slices given by ImagePositionPatient because of small gaps in the z-direction as mentioned in the model description above.

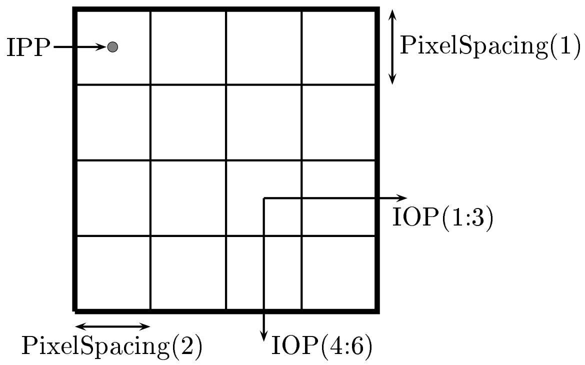

Figure 1: DICOM parameter definitions for a slice.

We have two different kinds of coordinate information, namely a patient based coordinate system (PCS) and an image based coordinate system (ICS). The ICS defines the discrete volume information $V(i_1, i_2, h)$ on a regular grid in the three dimensional space with grid points (x, y, z). Therefore, we can also write $V(x, y, z)$ instead of $V(i_1, i_2, h)$ and allocate the voxel indices (i_1, i_2, h) to volume coordinates $(x, y, z) \in \mathbb{R}^3$. $V(x, y, z)$ defines a voxel in PCS.

The PCS is defined by the above 5 variables, where IOP gives the directional cosines. We want to find a transformation matrix M between the two coordinate systems ICS and PCS. Without loss of generality we describe the transformation in an extended coordinate system (see Definition 1). The transformation matrix consists of rotation, translation and scaling whereas rotation is given by IOP, translation by IPP and scaling by PixelSpacing and IPP. We assume that IOP and PixelSpacing are equal between all slices H in a training data set. Furthermore we assume that the slices are

sorted and choose our global IPP candidate from the first slice.

The rotation matrix is defined by

$$R_{DCM} = \begin{pmatrix} dr & dc & ds & 0_3 \\ 0 & 0 & 0 & 1 \end{pmatrix} \tag{2}$$

where $0_3 = (0,0,0)^T$, $dr = (dr_x, dr_y, dr_z)^T = IOP(1:3)$ defines the directional cosines in row direction, $dc = (dc_x, dc_y, dc_z)^T = IOP(4:6)$ the directional cosines in column direction and $ds = (ds_x, ds_y, ds_z)^T = dr \times dc$ the directional cosines in slice direction whereas ds is defined by the cross product of dr and dc.

The scaling matrix is defined by

$$S_{DCM} = \begin{pmatrix} sc_x e_1 & sc_y e_2 & sc_z e_3 & 0_3 \\ 0 & 0 & 0 & 1 \end{pmatrix} \tag{3}$$

with $sc = (sc_x, sc_y, sc_z)^T = ext^{-1}(diag(S_{DCM}))$ and e_i is the 3×1 column vector with all elements being zeros except that the ith element is one. As mentioned above $sc_x = \frac{1}{l_x}$ and $sc_y = \frac{1}{l_y}$ are defined by PixelSpacing whereas sc_z is defined by the distance of IPP between two neighbor slices, e.g. the first and the second slice. Remember, we have assumed equidistant slices in a data set.

The translation matrix is defined by

$$T_t = \begin{pmatrix} e_1 & e_2 & e_3 & t \\ 0 & 0 & 0 & 1 \end{pmatrix} \tag{4}$$

where $t = (t_x, t_y, t_z)^T$ defines the translation vector.

The rotation, scaling and translation matrix provide us to transform between ICS and PCS.

Definition 2 (DICOM transformation matrix)**.** The DICOM transformation matrix is defined by

$$\Lambda_{DCM} = T_{IPP} R_{DCM} S_{DCM} T_0 \tag{5}$$

and transform an image coordinate $p^{im} = (i_1, i_2, h)^T$ to patient coordinate $p^p = (x, y, z)^T$ by $ext(p^p) = \Lambda_{DCM} ext(p^{im})$. T_0 is the translation which shift the slice to make top left voxel centered at $(0,0,0)$, i.e. $T_0 := T_t$ with $t = (-1,-1,-1)^T$. R_{DCM} and S_{DCM} are defined by (2) and (3) and T_{IPP} translate the top left voxel from the first slice at IPP.

Remark 1 (Inverse DICOM transformation matrix)*.* The inverse of Λ_{DCM} exist and transform a patient coordinate into an image coordinate by $ext(p^{im}) = \Lambda_{DCM}^{-1} ext(p^p)$.

Proof. The inverse of the rotation matrix is given by $R_{DCM}^{-1} = R_{DCM}^T$, the inverse of the translation matrix by $T_t^{-1} = T_{-t}$ and the inverse of scaling matrix by using $sc^{-1} = (\frac{1}{sc_x}, \frac{1}{sc_y}, \frac{1}{sc_z})$. □

Definition 3 (De-rotated patient coordinate system)**.** Corresponding to the PCS and the ICS we define a derotated patient coordinate system by $p^{dp} \in \mathbb{R}^3$ with $ext(p^{dp}) = R_{DCM}^T ext(p^p) = T_{IPP} S_{DCM} T_0 ext(p^{im})$. $IOP = (1,0,0,0,1,0)^T$ in a derotated PCS.

The are no rotational differences between the de-rotated PCS and the ICS. Furthermore, the volume and contour information are aligned to each other in the de-rotated PCS. Therefore, it is used for the statistical analysis.

2 Post-processing of BFE

This section discusses the reordering algorithm and introduce constraint and regularization parameters into our shape model. Let $n \in \{1, \dots, N\}$ be fixed in the following section and $l = 1, \dots, L$ the number of contour slices. We assume equidistant slice distances, well defined and sorted slices. Calculation are carried out inside ICS.

2.1 Reordering of the first and second principal axis

After the computation of $BFE_l \in \mathbb{R}^2 \times \mathbb{R}_+^2 \times (-\frac{\pi}{2}, \frac{\pi}{2}]$ we have to reorder the first and second principal axis to establish correspondence between the parameter of adjacent slices. The reordering algorithm is visualized in Figure 2 and given by the the following steps:

1. Transformation of the orientation parameter ϕ^l to directional cosines $\psi^l = (\cos\phi^l, sin\phi^l)^T$ to avoid computational non-uniqueness by the direction of the rotation.

2. Computation of the position of the basis slice by $M = \text{round}(\theta_3^1 + \frac{1}{2}(\theta_3^L - \theta_3^1))$ where round means rounding to the closest integer.

3. Initialization of reordering for the basis slice S_M. The first principal axis is chosen to be closest to $[1, 0]$ by determination of the distance of the x-axis to the first and second principal axis. The coordinate origin is defined by θ^M and the direction of x and y-axis are given by $(1,0)^T$ and $(0,1)^T$ in the ICS. Note that each principal axis can be defined in the opposite

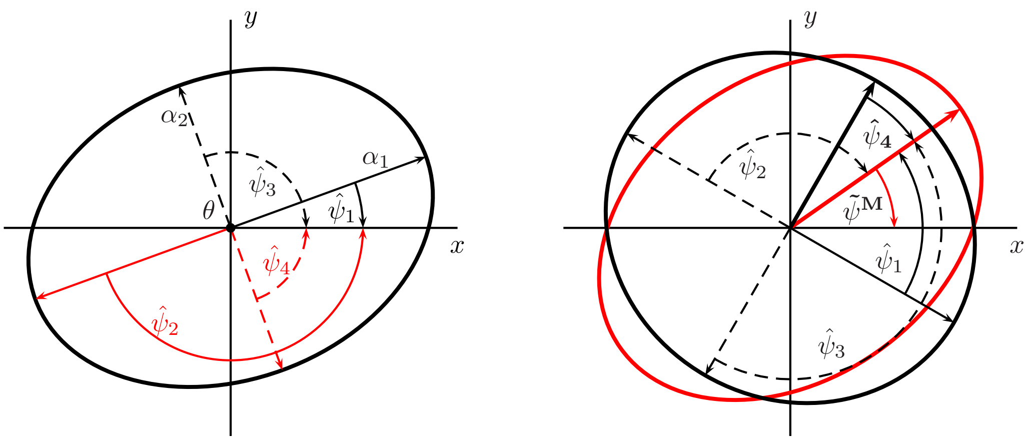

Figure 2: Visualization of the reordering procedure. Left image: Initialization of the reordering step for slice M. Right image: Reodering of slice $M-1$.

direction, i.e. the four possible directions are $\hat{\psi}_1^l = \psi^l$, $\hat{\psi}_2^l = -\psi^l$, $\hat{\psi}_3^l = (-sin\phi^l, \cos\phi^l)^T$ and $\hat{\psi}_4^l = -\hat{\psi}_3^l$. We obtain the reordered best fit $\underline{BFE}_l := (\theta^l, \tilde{\alpha}^l, \tilde{\psi}^l)^T$ by

$$\arccos((1,0)\tilde{\psi}^l) = \min_{i\in\{1,\ldots,4\}} \arccos((1,0)\hat{\psi}_i^l) \tag{6}$$

where $\hat{\psi}_i^l$ is a normal vector by definition and $l = M$.

4. Reordering from the basis slice to first slice, i.e. $l = M-1,\ldots,1$. The first principal axis $\tilde{\psi}^l$ is chosen to be closest to $\tilde{\psi}^{l+1}$ of the 4 axes defined by BFE_l, i.e., $\underline{BFE}_l$ is chosen to minimize $\arccos((\tilde{\psi}^{l+1})^T\hat{\psi}_i^l)$, $l = M-1,\ldots,1$.

5. Reordering from the basis slice to last slice, i.e., $l = M+1,\ldots,L$. The first principal axis $\tilde{\psi}^l$ is chosen to be closest to $\tilde{\psi}^{l-1}$ of the 4 axes defined by BFE_l, i.e., $\underline{BFE}_l$ is chosen to minimize $\arccos((\tilde{\psi}^{l-1})^T\hat{\psi}_i^l)$, $l = M+1,\ldots,L$.

Note, that $\underline{BFE}_l \in \mathbb{R}^2 \times \mathbb{R}_+^2 \times (-\pi,\pi]$ compared to $BFE_l \in \mathbb{R}^2 \times \mathbb{R}_+^2 \times (-\frac{\pi}{2},\frac{\pi}{2}]$. In fact, the rotation angle is not restricted by the above reordering procedure but we assume the maximal rotation between the basis and an arbitrary slice is $\max(|\phi^i - \phi^M|) = \pi, i \in \{1,\ldots,L\}$ after reordering. This allows a full twist of the prostate which we do not expect. In the following, we do not distinguish between BFE_l and $\underline{BFE}_l$. For the sake of convenience, we assume a reordered set of best-fitting ellipses and denote it simply by $BFE_l := (\theta^l, \alpha^l, \phi^l)^T \in \mathbb{R}^2 \times \mathbb{R}_+^2 \times (-\pi,\pi]$.

2.2 Constraint and relaxation

We introduce two additional constraints in the parameter model BFE_l, $l = 1, \dots, L$, which are relaxations of the rotation parameter ϕ in case of circularity and between large forward and backwards movings between neighbor slices.

Circularity: We define a cubic function $\lambda_l = c_l^p$ where $c_l = \frac{\min_l \alpha^l}{\max_l \alpha^l}$ is a measure of the circularity of the ellipse in slice $l = 1, \dots, L$ and p is a parameter which defines the power of the cubic function. The circularity is 1 if both axes have the same length. Simulations have shown that a choice of $p = 12$ leads to reasonable results in our model. The orientation parameter ϕ^l is relaxed by

$$\tilde{\phi}^l = \begin{cases} (1-\lambda_l)\phi^l, & l = M, \\ \lambda_l \phi^{l+1} + (1-\lambda_l)\phi^l, & l < M, \\ \lambda_l \phi^{l-1} + (1-\lambda_l)\phi^l, & l > M. \end{cases}$$

That means, in case of high circularity c_l we do not trust the orientation in the current slice l.

Relaxation between neighbor slices: Penalizing of large changes of the rotation between neighbor slices, i.e., between the parameters $\phi^{(l-1)}, \phi^l$ and $\phi^{(l+1)}$. At first, we note that the largest possible change between two slices is maximal $\frac{\pi}{4}$ after reordering as mentioned above. A simple quadratic function is used for the relaxation by

$$\tilde{\phi}^l = \begin{cases} \lambda_l \phi^{l+1} + (1-\lambda_l)\phi^l \text{ with } \lambda_l = \left(\frac{4}{\pi}|\phi^{l+1} - \phi^l|\right)^{\frac{1}{2}}, & l < M \\ \lambda_l \phi^{l-1} + (1-\lambda_l)\phi^l \text{ with } \lambda_l = \left(\frac{4}{\pi}|\phi^{l-1} - \phi^l|\right)^{\frac{1}{2}}, & l > M. \end{cases}$$

3 Control point methods

In this section, we present three Control Point (CP) methods with construction of the transformation matrix Λ_{dCP}^n and the parameter η^{nl}. The control point method used in the main article is described by CP3 in Section 3.3 (page S13). In addition, two alternatives are presented by method CP1 in Section 3.1 (page S8) and CP2 in Section 3.2 (page S11). Both sections can be skipped if the reader is only interested in CP3.

Before we construct a transformation matrix Λ_{dCP}^n between the derotated PCS and the sample space from the control points (see Figure 2 in the main article), we have to transform the control

points CP_n and ellipses parametrization BFE_{nl} from the ICS to the derotated PCS by $ext(p^{dp}) = \Xi ext(p^{im})$ with

$$\Xi = T_{IPP} S_{DCM} T_0$$

using Definition 3 in Section 1.3. Therewith, all data sets have the same origin and rotation of the underlying coordinate system between different data sets and same distances between points. Note, the voxel size can be different between different data sets. Therefore, distances between coordinates in the ICS might be different for different data sets. For reasons of simplicity, the mapping between the normal and the extended coordinate system is not mentioned explicit in the following section. In general, we have to carry out this step if we convert with the transformation matrix Ξ. The derotated prior data are given by the set $\{dBFE_{nl}, dCP_n\}$ with

$$dCP_n = \Xi(CP_n) \text{ and} \tag{7}$$

$$dBFE_{nl}^{pos} = (\Xi(p_1^{nl}), \Xi(p_2^{nl}), \Xi(p_3^{nl}))^T \tag{8}$$

whereas $BFE_{nl}^{pos} = (p_1^{nl}, p_2^{nl}, p_3^{nl})^T \in \mathbb{R}^9$ is the positional description of a best-fitting ellipse as described in the following remark.

Remark 2 (Positional BFE). An alternative representation for a best fitting ellipse is given by the set $BFE_{nl}^{pos} := (p_1^{nl}, p_2^{nl}, p_3^{nl})^T \in \mathbb{R}^9$ with

$$\begin{array}{rcl} p_1^{nl} & = & \theta^{nl}, \\ p_2^{nl} & = & \theta^{nl} + R(\alpha_1^{nl}, 0)^T, \\ p_3^{nl} & = & \theta^{nl} + R(0, \alpha_2^{nl})^T, \end{array}$$

$$\text{and } R = \begin{pmatrix} \cos\phi^{nl} & -\sin\phi^{nl} \\ \sin\phi^{nl} & \cos\phi^{nl} \end{pmatrix}$$

is a rotation matrix. We call this representation the positional best fitting ellipse. Both representations are isomorph to each other.

3.1 CP method 1

In the first approach, called CP1, we assume the definition of three control points $CP_n := (A^n, B^n, P_1^n) \in \mathbb{R}^{3\times 3}$. See Figure 3 for a visualization of the parameters which are defined as follow:

A^n centroid point of the prostate in the first contour slice of the prostate,

B^n centroid point of the prostate in the last contour slice of the prostate,

P_1^n point at the boundary of the prostate in posterior direction and in the base slice M (center slice, see Appendix 2.1).

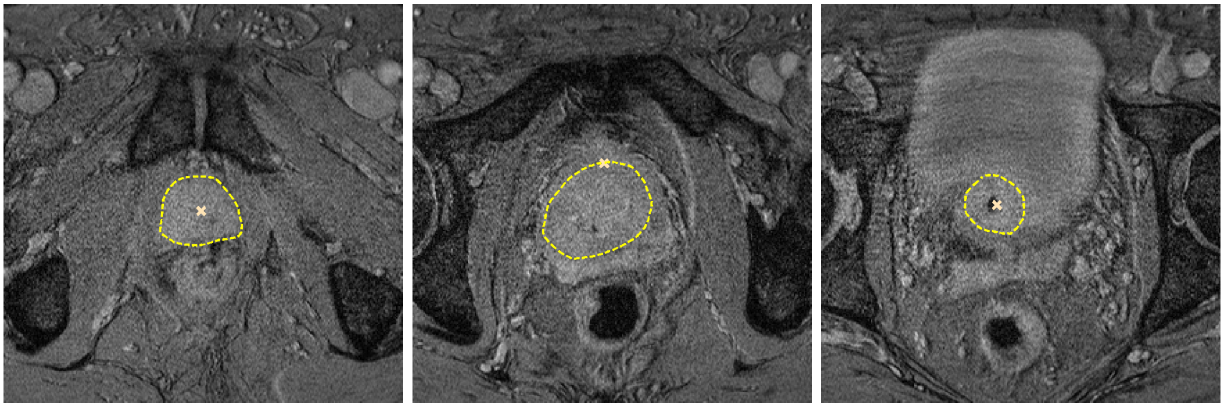

(a) A^n (b) P_1^n (c) B^n

Figure 3: Definition of control points using method CP1. The dashed line defines the manual delineation of the prostate. (a) Definition of A^n (first visible contour slice). (b) Definition of P_1^n in basis slice M. (c) Definition of B^n (last visible contour slice).

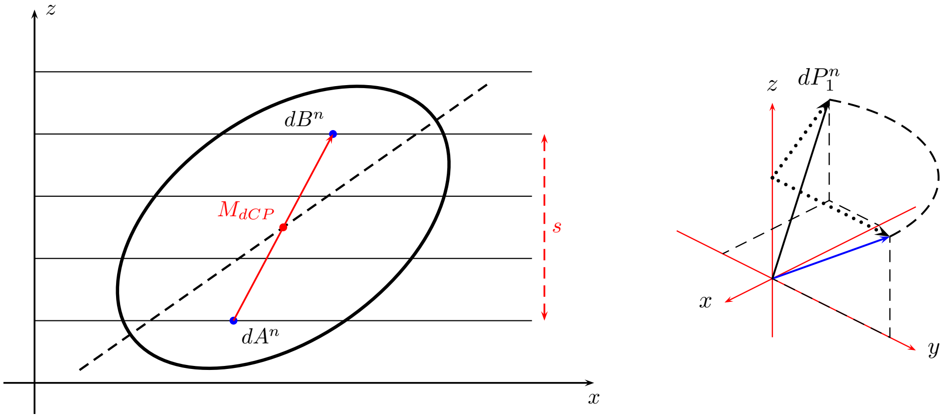

(a) (b)

Figure 4: (a) Visualization of translation point M_{CP} and the scaling s from the control points dA and dB by a cross section in slice direction z. (b) Rotation of dP_1 around the z-axis to a point with x-coordinate zero.

Given the derotated prior data we construct the transformation matrix Λ_{dCP}^n to a set of comparable best fitting ellipses for fixed n by the following steps (see Figure 4):

1. Translation of $M_{dCP_n} = \frac{1}{2}(dB^n - dA^n)$ to $(0,0,0)$ where $dCP_n := (dA^n, dB^n, dP_1^n)$. This results in the translation matrix $T_{-M_{dCP_n}}$ using formula (4) on page S4.

2. Rotation of the point dP_1^n around the z-axis with angle ζ given by the angle between the two vectors dP_1^n and $(0, 1, dP_1^n(z))^T$. The rotation matrix R_{dCP_n} is given by formula (1) on page S1 using $v = (0,0,1)^T$ and ζ as described before.

3. Scaling of the data by $s = |dB^n(z) - dA^n(z)|$. The scaling matrix is given by S_{dCP_n} with $sc = (\frac{\overline{L}-1}{s}, \frac{\overline{L}-1}{s}, \frac{\overline{L}-1}{s})^T$ using formula (3) on page S4 where $\overline{L}$ is defined as the common number of interpolated best fitting ellipses as described in section 2 of the main article. Note, we do not scale the length of the prostate by $\|dB^n - dA^n\|$ because we want to have the same length in z-direction. Figure 5 shows close correlation between both values for the training data set.

Finally, the transformation of the derotated prior data $\{dBFE_{nl}, dCP_n\}$ to $\{\overline{BFE}_{nl}, \overline{CP}_n\}$ is given by

$$\overline{BFE}_{nl}^{pos} = \left(\Lambda_{CP}^n(dp_1^{nl}), \Lambda_{CP}^n(dp_2^{nl}), \Lambda_{CP}^n(dp_3^{nl})\right)^T$$

and $\overline{CP}_n = \Lambda_{CP}^n(dCP^n)$ with

$$\Lambda_{dCP}^n = S_{dCP_n} R_{dCP_n} T_{-M_{dCP_n}}. \tag{9}$$

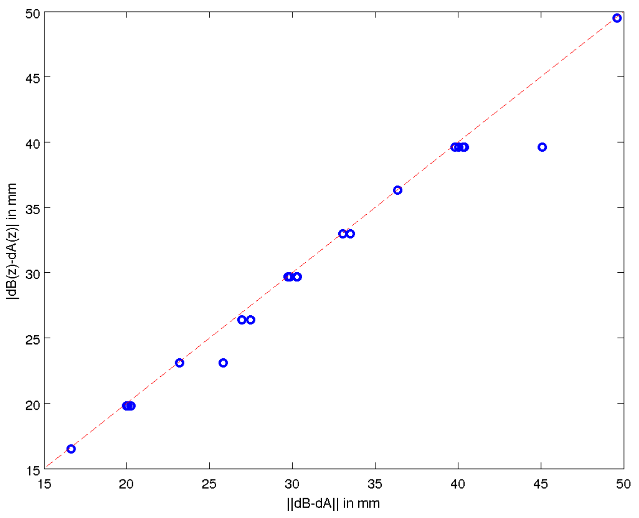

Figure 5: Comparison of the distance between the control points dA and dB in z-direction and $\|dB - dA\|$ (in mm). The dotted line shows equal values.

3.2 CP method 2

An alternative second approach, called CP2, assumes the definition of the control points $CP_n = (A^n, B^n, P_1^n, \ldots, P_{12}^n) \in \mathbb{R}^{3\times 14}, n = 1, \ldots, N$ with

A^n centroid point of the prostate in the first contour slice of the prostate,

B^n centroid point of the prostate in the last contour slice of the prostate,

$P_1^n, \ldots, P_{12}^n$ points at the boundary of the prostate in the base slice M whereas M is defined as described in Appendix 2.1.

Figure 6 visualizes the corresponding parameters plus the underlying manual delineation line which is not available in a new data set.

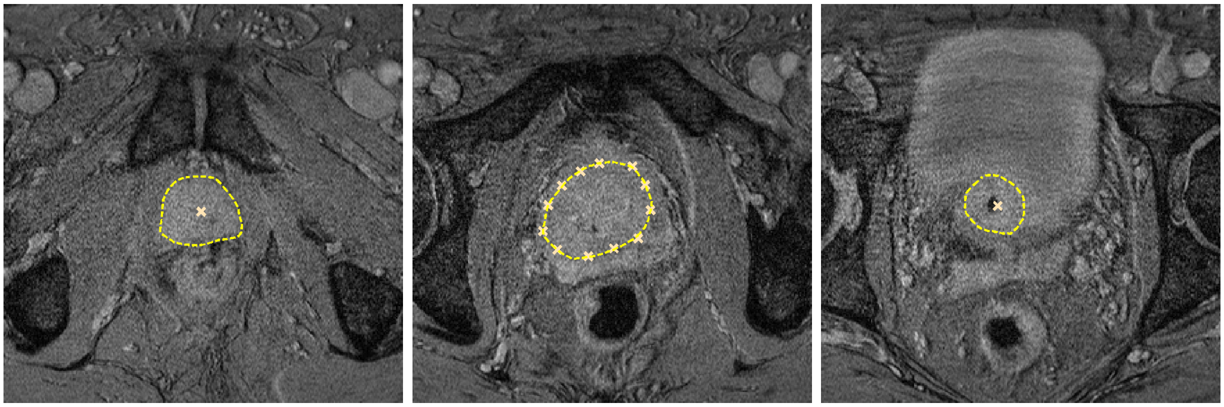

(a) A^n (b) P_m^n (c) B^n

Figure 6: Definition of control points using method CP2. The dashed line defines the manual delineation of the prostate. (a) Definition of A^n in the first visible contour slice. (b) Definition of P_m^n in basis slice M, $m = 1, \ldots, 12$. (c) Definition of B^n in the last visible contour slice.

Given the derotated prior data $\{dBFE_{nl}, dCP_n\}, l = 1, \ldots, L_n$ and $n = 1, \ldots, N$, we construct the transformation matrix Λ_{dCP}^n to a set of comparable best fitting ellipses for fixed n by the following steps:

1. Computation of the best fitting ellipse $CPBFE_n = (\theta^{CP_n}, \alpha^{CP_n}, \phi^{CP_n})^T \in \mathbb{R}^2 \times \mathbb{R}_+^2 \times (-\frac{\pi}{2}, \frac{\pi}{2}]$ for the set $\{P_1^n, \ldots, P_{12}^n\}$.
2. Transformation of $CPBFE_n$ to $dCPBFE_n = (\theta^{dCP_n}, \alpha^{dCP_n}, \phi^{dCP_n})^T$ in a derotated PCS.

3. The center slice is given by $s_n = \frac{L_n+1}{2}$ for each training data set n. If L_n is an odd number the center slice with the best-fitting ellipse is clearly defined. Otherwise, the best-fitting ellipses have to be interpolated to a number $L_n + 1$ before.

4. Estimation of the mean of the derotated best fitting ellipses in the center slice s_n from the training data, i.e.,

$$\mu^{dBFE} := (\mu_\theta^{dBFE}, \mu_\alpha^{dBFE}, \mu_\phi^{dBFE})^T = \frac{1}{N}\sum_n dBFE_{ns_n}. \quad (10)$$

Furthermore, we estimate the mean length by

$$\mu_L^{dBFE} = \frac{1}{N}\sum_n \|\theta^{dBFE_{nL_n}} - \theta^{dBFE_{n1}}\|$$

.

5. If L_n is an odd number then $M_{dCP_n} = \theta^{dCP_n}$. Otherwise M_{dCP_n} is determined from a cubic interpolation of the curve with support points in dA^n, θ^{dCP_n} and dB^n. The translation of $dCPBFE_n$ to the origin $(0,0,0)$ is given by the translation matrix $T_{-M_{dCP_n}}$ using formula (4) on page S4.

6. The rotation matrix R_{dCP_n} is given by formula (1) on page S1 using $v = (0,0,1)^T$ and $\zeta = -\phi^{dCP_n}$, i.e., we rotate the first principal axis of $dCPBFE_n$ to the direction of the x-axis.

7. Scaling of the data in x-direction by $sc_1 = \frac{\mu_{\alpha_1}^{dBFE}}{\alpha_1^{dCP_n}}$, y-direction by $sc_2 = \frac{\mu_{\alpha_2}^{dBFE}}{\alpha_2^{dCP_n}}$ and z-direction by $sc_3 = \frac{\mu_L^{dBFE}}{|dB^n(z) - dA^n(z)|}$. Thereby, the scaling matrix is given by S_{dCP_n} with $sc = (sc_1, sc_2, sc_3)^T$ using formula (3) on page S4. Note again, we do not scale the length of the prostate because we want to have the same length in z-direction.Figure 5 shows close correlation between both values for the training data set. Furthermore, Figure 7 illustrate the scaling parameters for our training data set and indicates correlations between them.

Finally, using CP method 2, the transformation of the derotated prior data $\{dBFE_{nl}, dCP_n\}$ to $\{\overline{BFE}_{nl}, \overline{CP}_n\}$ is given by

$$\overline{BFE}_{nl}^{pos} = \left(\Lambda_{dCP}^n(dp_1^{nl}), \Lambda_{dCP}^n(dp_2^{nl}), \Lambda_{dCP}^n(dp_3^{nl})\right)^T$$

and $\overline{CP}_n = \Lambda^n_{dCP}(dCP^n)$ with

$$\Lambda^n_{dCP} = S_{dCP_n} R_{dCP_n} T_{-M_{dCP_n}}. \tag{11}$$

An alternative to the above mentioned approach for computation of M_{dCP_n} is given by setting $M_{dCP_n} = \theta^{CP_n}$ for even and odd numbers L_n. Table 1 shows the average of the standard L1 and L2 norm of the distance vector between M_{dCP_n} and the interpolated center ellipse position from the corresponding training data. We argue for the implemented version using cubic interpolation due to a smaller error.

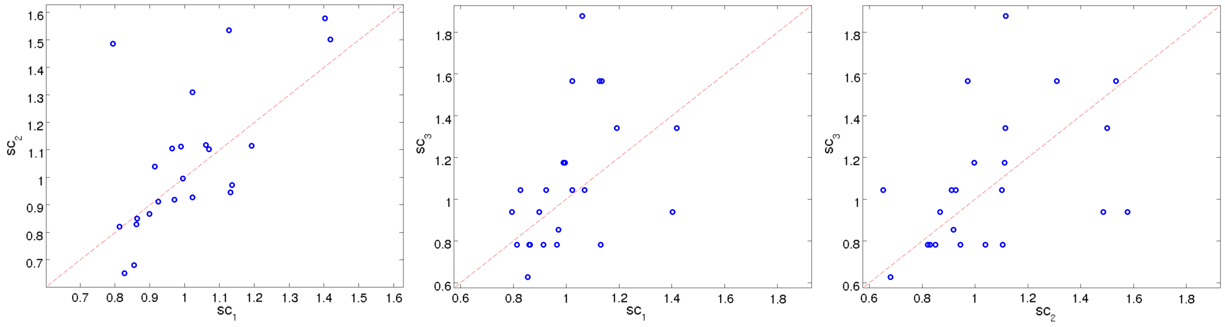

(a) sc_1 versus sc_2 (b) sc_1 versus sc_3 (c) sc_2 versus sc_3

Figure 7: Scaling parameters using transformation method 2 for the training data set.

Table 1: Average error of center ellipse position derived from control points versus center ellipse position derived from training data (unit: mm).

center ellipse position	alternative version	implemented version
$L1$ norm	0.5612	0.5470
$L2$ norm	0.4707	0.4568

3.3 CP method 3

The third approach, called CP3, is the primary used approach in this paper and assumes the definition of control points $CP_n = (A^n_{m_1}, B^n_{m_2}, P^n_{m_3}) \in \mathbb{R}^{3\times(m_1+m_2+m_3)}, m_i \in \mathbb{N}$ with

$A^n_{m_1}$ points at the boundary of the prostate in the first contour slice of the prostate,

$B^n_{m_2}$ points at the boundary of the prostate in the last contour slice of the prostate,

$P^n_{m_3}$ points at the boundary of the prostate in the base slice M (see subsection 2.1).

We have to assume $m_i \geq 3$ to be able to fit ellipses to the first, base and last slice. In the current work $m_1 = m_2 = m_3 = 6$ is chosen to achieve robust fits of best-fitting ellipses. See Figure 8 for a visualization of the parameters.

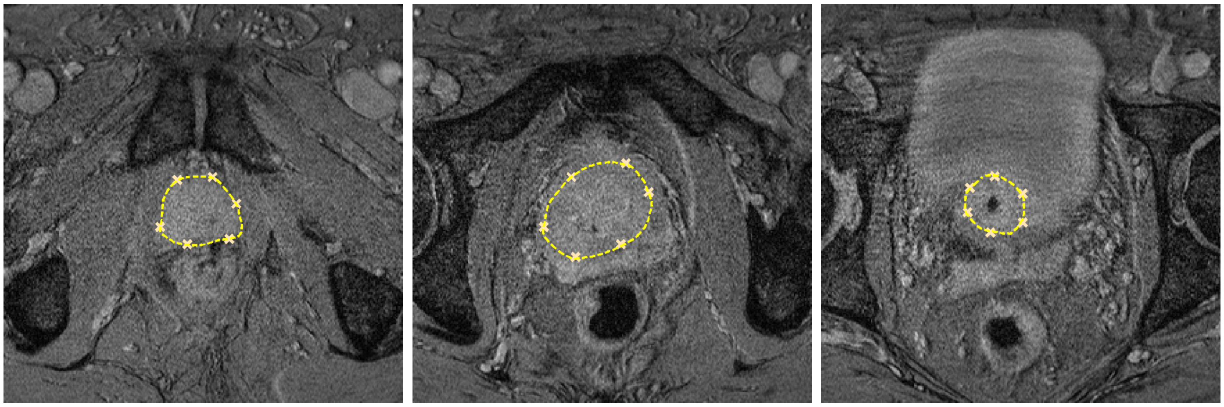

(a) $A^n_{m_1}$ (b) $P^n_{m_3}$ (c) $B^n_{m_2}$

Figure 8: Definition of control points at the boundary of the prostate using method CP3 with $m_1 = m_2 = m_3 = 6$. (a) Definition of $\{A^n_1, \ldots, A^n_{m_1}\}$ (first visible contour slice). (b) Definition of $\{P^n_1, \ldots, P^n_{m_3}\}$ in basis slice M. (c) Definition of $\{B^n_1, \ldots, B^n_{m_2}\}$ (last visible contour slice).

Given the derotated prior data $\{dBFE_{nl}, dCP_n\}$, we construct the transformation matrix Λ^n_{dCP} similar to Section 3.2. The main difference is the additional deformation of the ellipsoid by the control point information in the first and last slice. Let $n \in \{1, \ldots, N\}$ be fixed. Λ^n_{dCP} is derived by the following steps:

1. Computation of the best fitting ellipses $CPBFE_{n,i} = (\theta^{i,CP_n}, \alpha^{i,CP_n}, \phi^{i,CP_n})^T \in \mathbb{R}^2 \times \mathbb{R}^2_+ \times (-\frac{\pi}{2}, \frac{\pi}{2}], i = 1, 2, 3$ from the set $\{A^n_1, \ldots, A^n_{m_1}\}$ if $i = 1$, $\{B^n_1, \ldots, B^n_{m_2}\}$ if $i = 2$ and $\{P^n_1, \ldots, P^n_{m_3}\}$ if $i = 3$.

2. Transformation of $CPBFE_{n,i}$ to $dCPBFE_{n,i}$ in a derotated PCS with $dCPBFE_{n,i} = (\theta^{i,dCP_n}, \alpha^{i,dCP_n}, \phi^{i,dCP_n})^T$.

3. The center slice is given by $s_n = \frac{L_n+1}{2}$ for each training data set n. We assume that L_n is an odd number where the center slice with the best-fitting ellipse is clearly defined. Otherwise,

the set of derotated best-fitting ellipses $dBFE_n$ has to be interpolated to an odd number $L_n + 1$ before.

4. Estimation of mean $\mu^{i,dBFE}, i = 1,2,3$, from the derotated best-fitting ellipses in the first, last and center slice s_n from the training data by

$$\mu^{1,dBFE} := (\mu_\theta^{1,dBFE}, \mu_\alpha^{1,dBFE}, \mu_\phi^{1,dBFE})^T = \frac{1}{N}\sum_n dBFE_{n1},$$
$$\mu^{2,dBFE} := (\mu_\theta^{2,dBFE}, \mu_\alpha^{2,dBFE}, \mu_\phi^{2,dBFE})^T = \frac{1}{N}\sum_n dBFE_{nL_n},$$
$$\mu^{3,dBFE} := (\mu_\theta^{3,dBFE}, \mu_\alpha^{3,dBFE}, \mu_\phi^{3,dBFE})^T = \frac{1}{N}\sum_n dBFE_{ns_n}.$$

   Furthermore, we estimate the mean length by

$$\mu_L^{dBFE} = \frac{1}{N}\sum_n \|\theta^{dBFE_{nL_n}} - \theta^{dBFE_{n1}}\|$$

   .

5. If L_n is an odd number then $M_{dCP_n} = \theta^{3,dCP_n}$. Otherwise M_{dCP_n} is determined from a cubic interpolation of the curve with support points in θ^{1,dCP_n}, θ^{3,dCP_n} and θ^{2,dCP_n}. The translation of $dCPBFE_n$ to the origin $(0,0,0)$ is given by the translation matrix $T_{-M_{dCP_n}}$ using formula (4) on page S4.

6. The rotation matrix R_{dCP_n} is given by formula (1) on page S1 using $v = (0,0,1)^T$ and $\zeta = -\phi^{3,dCP_n}$, i.e., we rotate the first principal axis of $dCPBFE_{n,3}$ to the direction of the x-axis.

7. Determination of scaling values sc_{1l} and sc_{2l} of the first and second principal axis for slices $l = 1,\ldots,L_n$. At first, the scaling values in the first, center and last slice are calculated, i.e., $sc_{11} = \frac{\mu_{\alpha_1}^{1,dBFE}}{\alpha_1^{1,dCP_n}}$, $sc_{21} = \frac{\mu_{\alpha_2}^{1,dBFE}}{\alpha_2^{1,dCP_n}}$, $sc_{1L_n} = \frac{\mu_{\alpha_1}^{2,dBFE}}{\alpha_1^{2,dCP_n}}$, $sc_{2L_n} = \frac{\mu_{\alpha_2}^{2,dBFE}}{\alpha_2^{2,dCP_n}}$, $sc_{1s_n} = \frac{\mu_{\alpha_1}^{3,dBFE}}{\alpha_1^{3,dCP_n}}$ and $sc_{2s_n} = \frac{\mu_{\alpha_2}^{3,dBFE}}{\alpha_2^{3,dCP_n}}$. Afterwards, the remaining scaling values sc_{1l} and sc_{2l} are interpolated from $(sc_{11}, sc_{1s_n}, sc_{1L_n})$ and $(sc_{21}, sc_{2s_n}, sc_{2L_n})$. In addition, the scaling value in z-direction is determine by $sc_3 = \frac{\mu_L^{dBFE}}{|\theta_3^{2,dCP_n} - \theta_3^{1,dCP_n}|}$. Thereby, the scaling matrices are given by $S_{l,dCP_n}, l = 1,\ldots,L_n$, with $sc = (sc_{1l}, sc_{2l}, sc_3)^T$ using formula (3) on page S4. We do not scale the length of the prostate because we want to have the same length in z-direction. Figure 5 shows close correlation between both values for the training data set.

The primary used CP method 3 in this paper transforms the derotated prior data $\{dBFE_{nl}, dCP_n\}$ to $\{\overline{BFE}_{nl}, \overline{CP}_n\}$ by

$$\overline{BFE}_{nl}^{pos} = \left(\Lambda_{dCP}^n(dp_1^{nl}), \Lambda_{dCP}^n(dp_2^{nl}), \Lambda_{dCP}^n(dp_3^{nl})\right)^T$$

and $\overline{CP}_n = \Lambda_{dCP}^n(dCP^n)$ with

$$\Lambda_{dCP}^n := S_{l,dCP_n} R_{dCP_n} T_{-M_{dCP_n}}. \tag{12}$$

3.4 Description of θ^{nl} in relation to the control points

In addition to the position $\theta^{nl} = (\theta_1^{nl}, \theta_2^{nl}, \theta_3^{nl})^T$ of each best-fitting ellipse, we describe the parameter in terms of a distance vector η^{nl} of θ^{nl} to a line or a curve, defined by the control points. Thereby, we are describing the mean shape which is closest to the control points. Corresponding to the different control point methods, see sections before, there are different ways of describing the positional parameter via η^{nl}. Let $n = 1, \ldots, N$ be fixed in this section.

3.4.1 Determination of η^{nl} using CP method 1

Given $dCP_n := (dA^n, dB^n, dP_1^n)$ the two control points dA^n and dB^n define a line $\overrightarrow{dA^n dB^n} \subset \mathbb{R}^3$ that cross each slice $l = 1, \ldots, L_n$ with prostate contour information. Now, κ^{nl} defines the intersection point of the line $\overrightarrow{dA^n dB^n}$ with each slice l. It is given by $\kappa^{nl} = dA^n + \frac{\tilde{l}}{L_n - 1} v^n$ with $\tilde{l} = l - 1$, $v^n = dB^n - dA^n$ and $l \in \{0, \ldots, L_n\}$. Thereby, the distance vector η^{nl} to θ^{nl} is defined by

$$\eta^{nl} = \theta^{nl} - \kappa^{nl},$$

with $l \in \{0, \ldots, L_n\}$. Simulations have shown that statistical inference of η^{nl} result in a much smaller variance than for θ^{nl}.

3.4.2 Determination of η^{nl} using CP method 2 and 3

Instead of construction a line $\overrightarrow{dA^n dB^n}$ as described before, we construct a cubic curve that connect three slices with contour points, i.e., the first, the basis and the last slice. Using CP method 2, dCP_n is defined by

$$dCP_n := (dA^n, dB^n, dP_1^n, \ldots, dP_{12}^n)$$

and $dCPBFE_n = (\theta^{dCP_n}, \alpha^{dCP_n}, \phi^{dCP_n})$. The three points dA^n, θ^{dCP_n} and dB^n define a curve $g_{dCP}(z)$. Similar, in case of CP method 3, $CP_n := (dA^n_1, \ldots, dA^n_{m_1}, dB^n_1, \ldots, dB^n_{m_2}, dP^n_1, \ldots, dP^n_{m_3})$ results in the three best fitting ellipses $dCPBFE_{n,i} = (\theta^{i,dCP_n}, \alpha^{i,dCP_n}, \phi^{i,dCP_n}), i = 1, 2, 3$. Therewith, the three points θ^{i,dCP_n} define a cubic curve $g_{dCP}(z)$. The final curve is calculated by cubic interpolation with supporting points $\{A^n, \theta^{dCP_n}, B^n\}$ for CP2 or $\{\theta^{1,dCP_n}, \theta^{3,dCP_n}, \theta^{2,dCP_n}\}$ for CP3. Therewith, $g_{dCP}(z)$ cross each slice, whereas each slice is defined by an (x, y, z)-plane with $z = \theta^{nl}_3, l = 1, \ldots, L_n$. Let $g_{CP}(\theta^{nl})$ define the intersection points of the curve $g_{CP}(z)$ with each slice. The distance vector η^{nl} to θ^{nl} is calculated by

$$\eta^{nl} = \theta^{nl} - g_{CP}(\theta^{nl})$$

with $l \in \{0, \ldots, L_n\}$.

Figure 9 and 10 show μ^l_θ, μ^l_η, θ^{nl} and η^{nl} of the training data set in the sample space using CP3 with $l = 1, \ldots, 23$. The covariance matrices are visualized by 90% confidence ellipses. We can clearly decrease the variance using the alternative approach with identical variance for the base slice because of $\kappa^{nl} = 0 \quad \forall n = 1, \ldots, N$ for $l = M$.

4 Posterior distribution

In addition to construct a shape model and the corresponding shape space, we aim to estimate the best fitting ellipses BFE_l parametrized by $\rho_l = (\theta^l, \alpha^l, \phi^l)^T$, $l = l, \ldots, L$ in a new observed data set given a set of control points CP. In general, this can be obtained through the posterior $\pi(\rho \mid S)$ where $s_{il} \in S \subseteq V$ is the volume information and $i = (i_1, i_2) \in I(\rho)$ is a set of indices within the ellipses ρ. The control points CP are used to deform the prior model $\pi(\rho)$ as described in Section 3. Therefore, we model the posterior by an empirical Bayes approach [2]. The posterior

$$\pi(\rho \mid S, CP) \propto L(S \mid \rho) * \pi(\rho \mid CP)$$

defines the posterior density of the deformed template $\pi(\rho \mid CP)$ given the the observed image. The likelihood or image model $L(S \mid \rho)$ is the joint probability density function of the gray levels given the parametrized object $\rho|_{CP}$, whereas $\rho|_{CP}$ defines the ellipses ρ deformed by the control points CP. The prior $\pi(\rho \mid CP)$ models realistic variations from our mean shape $\mu_{BFE} \in (\mathbb{R}^2 \times \mathbb{R}^2_+ \times (-\pi, \pi])^L$ given the control points.

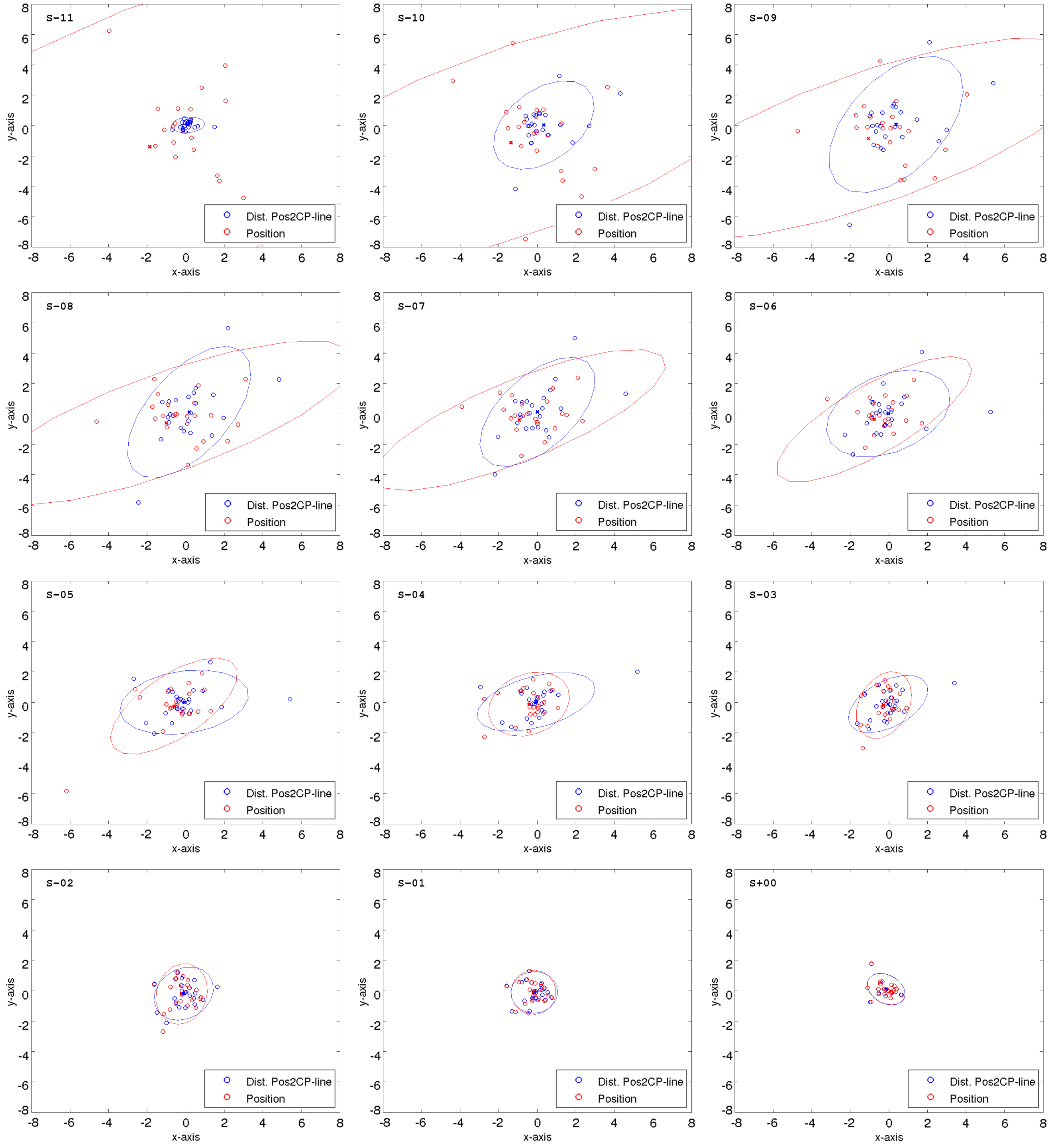

Figure 9: Slice S $= -11, \ldots, 0$ where 0 mark the base-slice (i.e. $-\frac{L-1}{2}, \ldots, 0$). Each plot contains 23 red and blue circles representing θ^{nl} (Position) and η^{nl} (Dist. Pos2CP-line) respectively, where $N = 23$ is the number of training cases and $n = 1, \ldots, N$. The means μ_θ^l and μ_η^l are depicted by small crosses. The corresponding covariance matrices are depicted by 90% confidence ellipses.

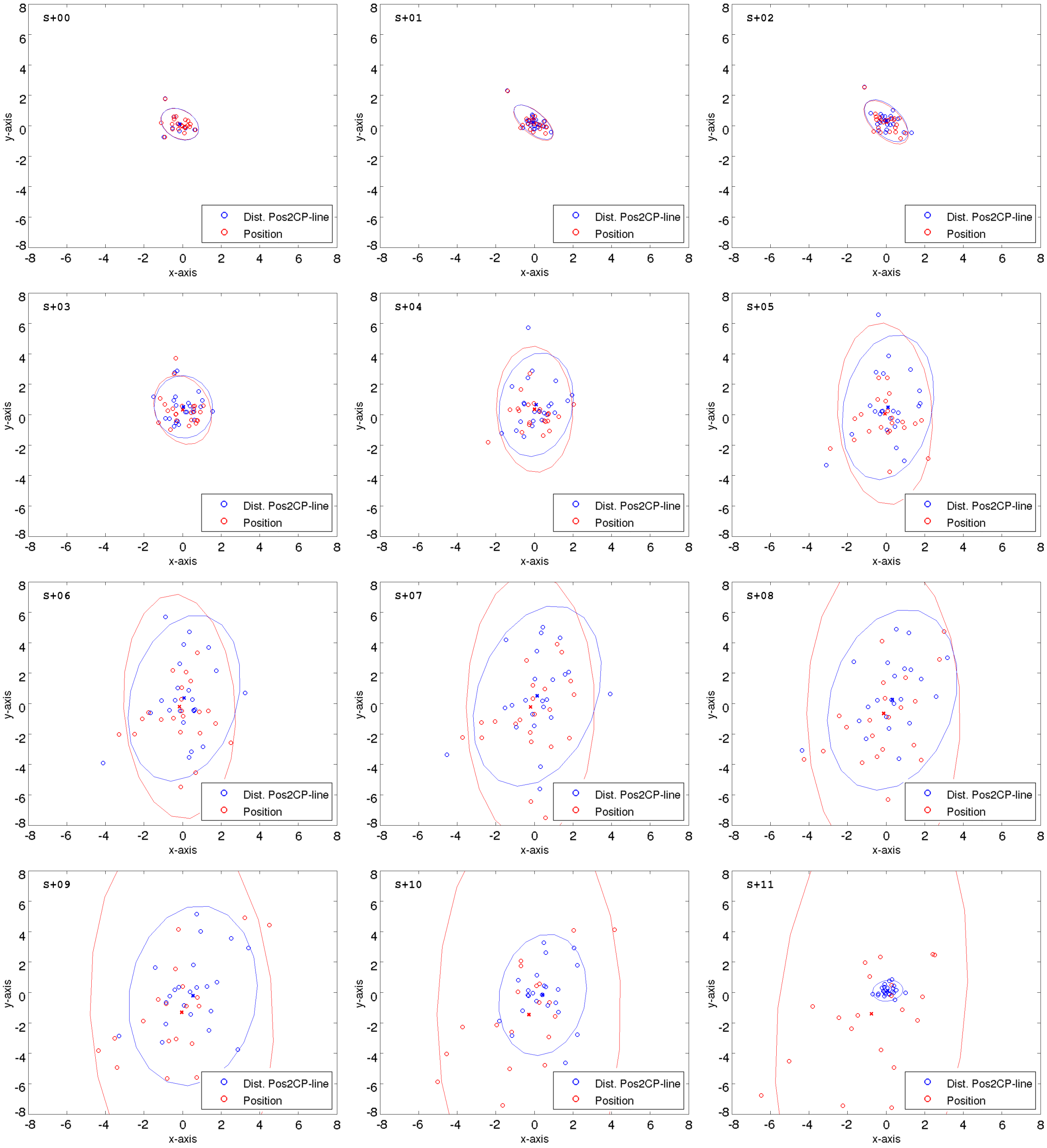

Figure 10: Slice S $= 0, \ldots, 11$ where 0 mark the base-slice (i.e. $0, \ldots, \frac{L-1}{2}$). Each plot contains 23 red and blue circles representing θ^{nl} (Position) and η^{nl} (Dist. Pos2CP-line) respectively, where $N = 23$ is the number of training cases and $n = 1, \ldots, N$. The means μ_θ^l and μ_η^l are depicted by small crosses. The corresponding covariance matrices are depicted by 90% confidence ellipses.

We assume different mean image gray values ν^0 inside and ν^1 outside of the object and constant variance τ^2 as proposed in Dryden and Mardia [3]. Therewith, the likelihood can be modeled by

$$L(S \mid \rho, CP) \propto \exp\left(-\frac{1}{2\tau^2} \sum_{l=1}^{L} \sum_{i \in I(\rho_l|_{CP})} (s_{il} - \nu^0)^2 \right.$$
$$\left. -\frac{1}{2\tau^2} \sum_{l=1}^{L} \sum_{i \notin I(\rho_l|_{CP})} (s_{il} - \nu^1)^2 \right)$$

with $s_i = \nu^0 + \epsilon_i$ if $i \in I(\rho|_{CP})$ and $s_i = \nu^1 + \epsilon_i$ if $i \notin I(\rho|_{CP})$ and an error distribution of $\epsilon_i \overset{\text{iid}}{\sim} N(0, \tau^2)$.

Using the suggest prior distribution from section 2 in the main article, the prior $\pi(\rho \mid CP) = \pi(\theta, \alpha, \phi \mid CP)$ is given by

$$\pi(\rho \mid CP) \propto \exp\left(-\beta \sum_l \frac{1}{2(\sigma^l_{\theta_1})^2} (\theta^l_1 - \mu^l_{\theta_1})^2 - \beta \sum_l \frac{1}{2(\sigma^l_{\theta_2})^2} (\theta^l_2 - \mu^l_{\theta_2})^2 \right)$$
$$* \exp\left(-\beta \sum_l \frac{\mu^l_{\alpha_1}}{2(\sigma^l_{\alpha_1})^2} (\alpha^l_1 - \ln(\mu^l_{\alpha_1}))^2 \right)$$
$$* \exp\left(-\beta \sum_l \frac{\mu^l_{\alpha_2}}{2(\sigma^l_{\alpha_2})^2} (\alpha^l_2 - \ln(\mu^l_{\alpha_2}))^2 \right)$$
$$* \exp\left(-\beta \sum_l \frac{1}{2(\sigma^l_{\phi})^2} (\phi^l - \mu^l_{\phi})^2 \right) \Bigg|_{CP}$$

with $\theta^l_i = \xi^l_i + \eta^l_i, i = 1, 2$ (see section 3.4). The weight parameter $\beta \in \mathbb{R}_+$ describes the importance of the prior compared to the likelihood. In addition, we extend the prior by taking into account the variation between neighbor slices by

$$\pi_1(\rho \mid CP) \propto \pi(\rho \mid CP)$$
$$* \exp\left(-\tilde{\beta}_1 \sum_{i=1,2} \sum_l (\theta^{l-1}_i - \theta^l_i)^2 + (\theta^{l+1}_i - \theta^l_i)^2 \right)$$
$$* \exp\left(-\tilde{\beta}_2 \sum_{i=1,2} \sum_l (\alpha^{l-1}_i - \alpha^l_i)^2 + (\alpha^{l+1}_i - \alpha^l_i)^2 \right)$$
$$* \exp\left(-\tilde{\beta}_3 \sum_l (\phi^{l-1} - \phi^l)^2 + (\phi^{l+1} - \phi^l)^2 \right) \Bigg|_{CP},$$

where $\tilde{\beta}_1 = \beta\beta_1, \tilde{\beta}_2 = \beta\beta_2, \tilde{\beta}_3 = \beta\beta_3$ and $\beta_1, \beta_2, \beta_3 \in \mathbb{R}_+$ are regularization parameters which have to be chosen. Therewith, we introduce a constraint that small changes between neighbor slices are

more likely than jumps. An alternative approach is to take into account neighbor relations by the second derivatives, i.e.,

$$\begin{aligned}\pi_2(\rho \mid CP) \propto \pi(\rho \mid CP) \\ &* \exp\left(-\tilde{\beta}_1 \sum_l (\theta_1^{l+1} - 2\theta_1^l + \theta_1^{l-1})^2 + (\theta_2^{l+1} - 2\theta_2^l + \theta_2^{l-1})^2\right) \\ &* \exp\left(-\tilde{\beta}_2 \sum_l (\alpha_1^{l+1} - 2\alpha_1^l + \alpha_1^{l-1})^2 + (\alpha_2^{l+1} - 2\alpha_2^l + \alpha_2^{l-1})^2\right) \\ &* \exp\left(-\tilde{\beta}_3 \sum_l (\phi^{l+1} - 2\phi^l + \phi^{l-1})^2\right)\Bigg|_{CP}\end{aligned}$$

with $\tilde{\beta}_1, \tilde{\beta}_2, \tilde{\beta}_3$ as before. Hence, the posterior becomes

$$\pi(\rho \mid S, CP) \propto L(S \mid \rho) * \pi_i(\rho \mid CP), \quad i \in \{1, 2\}. \tag{13}$$

Estimation of $\pi(\rho \mid S, CP)$ can be done by Markov chain Monte Carlo (MCMC) method using the Metropolis-Hastings algorithm [4]. The MCMC approach avoids the computation of the unknown normalization constant in the posterior $\pi(\rho \mid S, CP)$ by drawing independent samples from a proposal distribution and the generation of a Markov chain. The MCMC method is implemented as follows:

1. Choosing of an arbitrary initial estimate of $\rho|_{CP}$, e.g, by the prior model.

2. Generation of a random permutation $\mathbb{I}_p = \{i_1, \ldots, i_L\}$ from the index set $\{1, \ldots, L\}$ whereas L is the number of slices. Therewith, we update the slices in a random order.

3. Generation of a new random realization from the proposal distribution $g_(\rho_{new,i_k} \mid \rho_{old,i_k})$, e.g., by $\rho_{new,i_k} = (\theta^{new,i_k}, a^{new,i_k}, \phi^{new,i_k})^T \sim N(\rho_{old,i_k}, \chi^2_{i_k})$ where $\chi^2_{i_k}$ can be chosen depending on the prior distribution by $\chi_{i_k} = diag(\lambda_1\sigma^{i_k}_{\theta_1}, \lambda_2\sigma^{i_k}_{\theta_2}, \lambda_3\sigma^{i_k}_{a_1}, \lambda_4\sigma^{i_k}_{a_2}, \lambda_5\sigma^{i_k}_{\phi})$, $k = 1, \ldots, L$ where

λ_i, $i = 1, \ldots, 5$ are weights that must be chosen. We assume a density

$$g(\rho_{new,i_k} \mid \rho_{old,i_k}) \propto \exp\left(-\frac{1}{2}\sum_{j=1}^{2}\frac{1}{(\sigma_{\theta_j}^{i_k})^2}(\theta_j^{new,i_k} - \theta_j^{old,i_k})^2 - \frac{1}{2}\sum_{j=1}^{2}\frac{1}{(\sigma_{a_j}^{i_k})^2}(a_j^{new,i_k} - a_j^{old,i_k})^2 - \frac{1}{2}\sum_{j=1}^{2}\frac{1}{(\sigma_{\phi_j}^{i_k})^2}(\phi_j^{new,i_k} - \phi_j^{old,i_k})^2\right).$$

4. Calculation of the Hastings-ratio

$$p = \frac{\pi(\rho_{new} \mid S, CP)g(\rho_{old} \mid \rho_{new})}{\pi(\rho_{old} \mid S, CP)g(\rho_{new} \mid \rho_{old})}$$

   using the derived formula 13 for the posterior density. Note, the used proposal density is symmetric and therefore

$$p = \frac{\pi(\rho_{new} \mid S, CP)}{\pi(\rho_{old,k} \mid S, CP)}.$$

5. Accept $\rho_{i_k} = \rho_{new,i_k}$ with probability $min(1, p)$ otherwise keep $\rho_{i_k} = \rho_{old,i_k}$.

6. Repeat steps 3 to 5 L-times until each slice is updated.

Typically the MCMC method consist of a burn-in and a sample period by an iteration of steps 2 to 6. The final $\rho|_{CP}$ is calculated by the average of the sample period. In our simulations we used a burn-in and sample period of 500 samples each. Furthermore, calculations are done inside a mask around the registered prior shape to decrease computation time and to avoid additional variance by other structures in the lower male pelvis.

5 Additional data analysis

This section reports additional results for the prior as well as for the MCMC method described in Section 4 before.

5.1 Prior results

In Section 3 different approaches of control points are discussed. We obtain a different mean shape as a prior corresponding to the used method. The results in Section 3 in the main article assume

the control point method CP3. Alternatively, Table 2 shows the the evaluation results between the manual delineation and the deformed MBFE to the manual contours for each test data set using CP2. All evaluation criterias in Table 3 (in the main article) show a better performance than in Table 2. We do not show the results of control point method CP1 here because the results are even less accurate than for CP2. Even if we observe a less accurate performance of CP2 compared to CP3, the method has the advantage of fewer control points in the outer slices where the definition of the delineation is more challenging. Therefore, this method is an object of further studies.

Table 2: CP2: Evaluation metrics between Mean Best Fitting Ellipse and manual delineations given by the physician. HD mean is given in mm, Dice 3D and Accuracy in percentage.

Test set	1	2	3	4	5	6	7	8	9	10	μ
HD mean	4.58	2.77	6.32	3.61	4.12	4.66	3.43	6.82	4.03	4.04	4.44
Dice 3D	0.83	0.90	0.85	0.87	0.87	0.83	0.89	0.84	0.92	0.89	0.87
Accuracy	0.69	0.81	0.74	0.76	0.73	0.63	0.80	0.64	0.84	0.78	0.74

5.2 MCMC results

In this section we discuss results after MCMC as described in Section 4 with $\pi_2(\rho \mid CP)$ as the prior distribution whereas we assume control points method CP3 as described in Section 3.3. In the case of CP3, we do not update the first, center and last slice because the underlying contour is described reasonable well by the control point.

The weight β and regularization parameters β_1, β_2 and β_3 of the prior distribution have to be chosen in practice. A simulation study (reported below) motivates, e.g., a choice of $\beta = 40$ and $(\beta_1, \beta_2, \beta_3) = (2, 180, 10)$.

Figures 11 and 12 depict the influence of different β, β_1, β_2 and β_3 in formula (13) by a comparison of the distances (7)-(9) as described in the main article. $\beta = 0$ means that the posterior is only driven by the likelihood. On the other hand, $\beta \to \infty$ means we ignore the likelihood and the posterior is only determined by the prior. We can observe the volume overlap and accuracy converged against the prior for increasing weights β. The regularization parameters β_1, β_2 and β_3 control the speed of the convergence to the prior (MBFE) and stabilize the results.

Based on initial simulations with several parameter combinations, we replicated 200 times the MCMC step for 3 different patients from the test data set with $\beta \sim U(0, 100)$, $\beta_1 \sim U(0, 20)$,

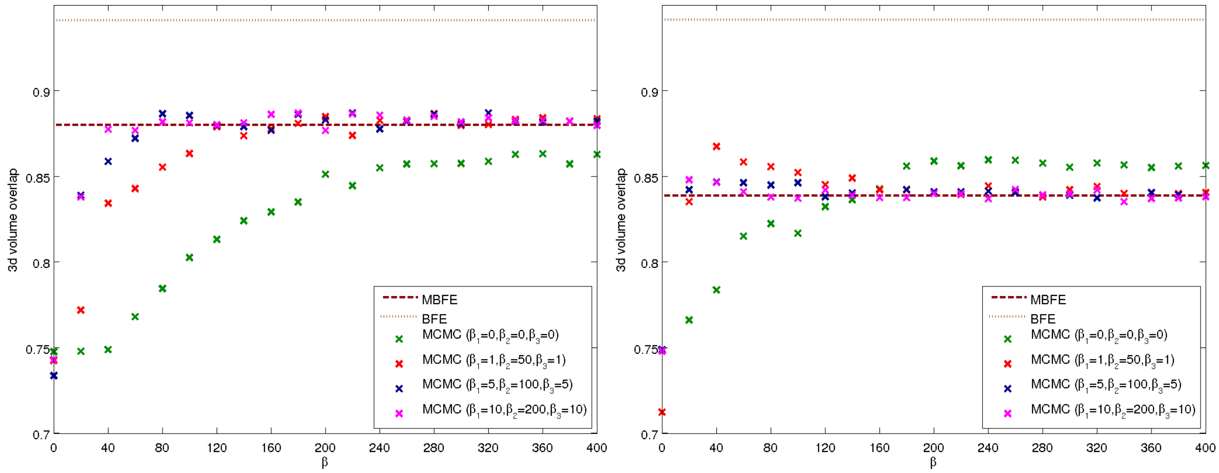

(a) test data set 5 (b) test data set 6

Figure 11: Volume overlap after MCMC using different $(\beta, \beta_1, \beta_2, \beta_3)$ settings for two different test data sets compared to the volume overlap of BFE and MBFE with manual delineation line.

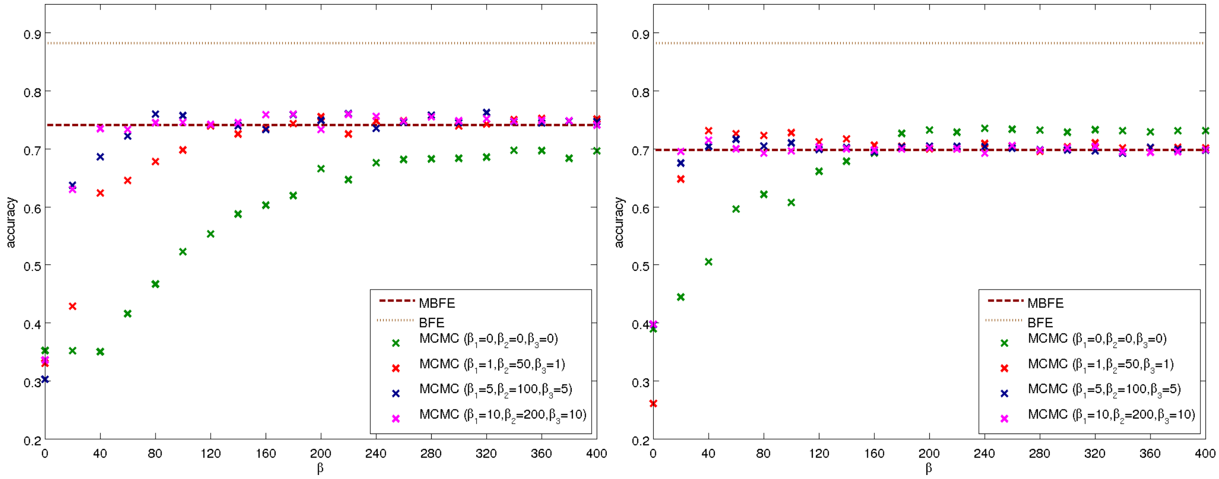

(a) test data set 5 (b) test data set 6

Figure 12: Accuracy after MCMC using different $(\beta, \beta_1, \beta_2, \beta_3)$ settings for two different test data sets compared to the accuracy of BFE and MBFE with manual delineation line.

$\beta_2 \sim U(0, 200)$ and $\beta_3 \sim U(0, 30)$ where $U(a, b)$ denote the standard uniform distribution on the open interval (a, b). The results with the two highest dice coefficients (volume overlap) are shown in Table 3.

On the basis of the simulation results we have chosen five different MCMC parameter settings $M_i = \{\beta, \beta_1, \beta_2, \beta_3\}$ by $M_1 = \{94, 6, 181, 28\}$, $M_2 = \{32, 1.6, 173, 1\}$, $M_3 = \{53, 1.1, 140, 12\}$, $M_4 = \{40, 2, 180, 10\}$ and $M_5 = \{80, 10, 180, 20\}$. Tables 4, 5 and 6 illustrate the distances between

Table 3: Prior distribution parameters ranked by the two highest dice 3D coefficients after 200 simulations for 3 different test data sets.

Test data set	Weights for the prior distribution β	$\tilde{\beta}_1$	$\tilde{\beta}_2$	$\tilde{\beta}_3$	Dice 3D	Accuracy
5	94.08	15.95	181.13	27.92	0.89	0.77
	92.17	13.22	118.95	10.91	0.89	0.76
6	53.64	1.14	138.84	12.48	0.87	0.74
	47.42	1.10	27.69	5.27	0.86	0.73
8	32.36	1.62	173.12	0.95	0.90	0.80
	28.31	1.75	181.46	19.35	0.90	0.80

the manual delineation and the MCMC result for each test data set and the five different parameter sets. For comparison M_0 shows the results from the registered mean shape without applying MCMC as we have seen in Table 2 and 3 in the main article. A comparison with the results indicate that we do not gain large improvement by MCMC compared to the registered mean shape using the control points. In fact, the 10 test data result in a median volume overlap and accuracy of 0.90, 0.81 for M_0 (MBFE) compared to 0.91, 0.82 for the parameter set M_4 (MCMC), with a median absolute deviation (MAD) of $0.021, 0.035$ compared to $0.021, 0.040$. The values show a slight overall improvement after MCMC. Particularly, there is an improvement of the accuracy in Table 6 for all patients in case of parameter set M_5. Furthermore, test data set 9 in Table 5 shows a significant improvement of the volume overlap after MCMC. The slices of this patient are depicted in Figure 13 together with the manual contour information, contour line from deformed mean shape and contour line after MCMC. Nevertheless, we believe the MCMC procedure is a too time consuming step in the praxis compared to the deformation of the mean shape given control points.

Table 4: HD mean between MCMC results and manual delineations given by the physician using different parameter sets $M_i = \{\beta, \tilde{\beta}_1, \tilde{\beta}_2, \tilde{\beta}_3\}$ (in mm).

	Test data set 1	2	3	4	5	6	7	8	9	10	md	MAD
M_0	2.51	2.17	4.24	2.83	3.75	4.63	1.95	4.67	4.27	4.42	4.00	0.655
M_1	2.26	1.95	4.38	2.96	4.02	4.63	1.93	4.58	2.91	4.47	3.49	1.034
M_2	2.18	1.96	4.63	2.88	4.65	4.63	1.89	4.34	3.88	5.00	4.11	0.719
M_3	2.26	2.05	4.81	2.99	4.09	4.63	1.90	4.75	3.68	4.59	3.88	0.883
M_4	2.17	2.15	4.43	3.07	4.36	4.63	1.71	4.33	4.01	4.53	4.17	0.413
M_5	2.19	1.92	4.12	2.75	4.03	4.63	1.93	4.67	2.58	4.46	3.39	1.136

To verify further the proposed posterior distribution in formula (13), we compare the volume

Table 5: Dice 3D between MCMC results and manual delineations given by the physician using different parameter sets $M_i = \{\beta, \beta_1, \beta_2, \beta_3\}$ (in percentage).

	Test data set											
	1	2	3	4	5	6	7	8	9	10	md	MAD
M_0	0.92	0.93	0.90	0.91	0.88	0.84	0.92	0.88	0.89	0.88	0.90	0.021
M_1	0.93	0.93	0.91	0.90	0.88	0.84	0.91	0.88	0.92	0.88	0.91	0.023
M_2	0.93	0.93	0.90	0.91	0.84	0.85	0.91	0.90	0.92	0.87	0.90	0.026
M_3	0.93	0.93	0.91	0.91	0.88	0.86	0.90	0.89	0.92	0.88	0.90	0.023
M_4	0.93	0.93	0.91	0.90	0.87	0.85	0.91	0.89	0.92	0.88	0.91	0.021
M_5	0.93	0.93	0.91	0.90	0.88	0.84	0.91	0.88	0.93	0.89	0.90	0.021

Table 6: Accuracy between MCMC results and manual delineations given by the physician using different parameter sets $M_i = \{\beta, \beta_1, \beta_2, \beta_3\}$ (in percentage).

	Test data set											
	1	2	3	4	5	6	7	8	9	10	md	MAD
M_0	0.84	0.84	0.81	0.82	0.74	0.70	0.84	0.73	0.80	0.74	0.81	0.035
M_1	0.86	0.86	0.82	0.81	0.75	0.70	0.83	0.74	0.85	0.75	0.82	0.047
M_2	0.87	0.86	0.80	0.83	0.64	0.72	0.82	0.80	0.85	0.71	0.81	0.046
M_3	0.86	0.85	0.81	0.83	0.73	0.74	0.81	0.79	0.85	0.74	0.81	0.042
M_4	0.87	0.86	0.82	0.82	0.71	0.73	0.84	0.78	0.85	0.75	0.82	0.040
M_5	0.86	0.86	0.82	0.82	0.75	0.71	0.84	0.76	0.86	0.76	0.82	0.047

overlap and accuracy in Figure 14 between MCMC results and MBFE result based on different volume information. The MCMC results are calculated for different β, fixed $(\beta_1, \beta_2, \beta_3) = (2, 180, 10)$ and on the basis of:

1. the original volume information,
2. a derived volume from the original by setting all gray values inside the manual delineation line to 100 and outside to 130 added by some error with the same variance inside and outside,
3. a derived volume from the original by setting all gray values inside the best fitting ellipse to 100 and outside to 130 added by some error with the same variance inside and outside,
4. a derived volume from the original by setting all gray values inside the manual delineation line to 80 and outside to 150 added by some error with the same variance inside and outside,
5. a derived volume from the original by setting all gray values inside the best fitting ellipse to 80 and outside to 150 added by some error with the same variance inside and outside.

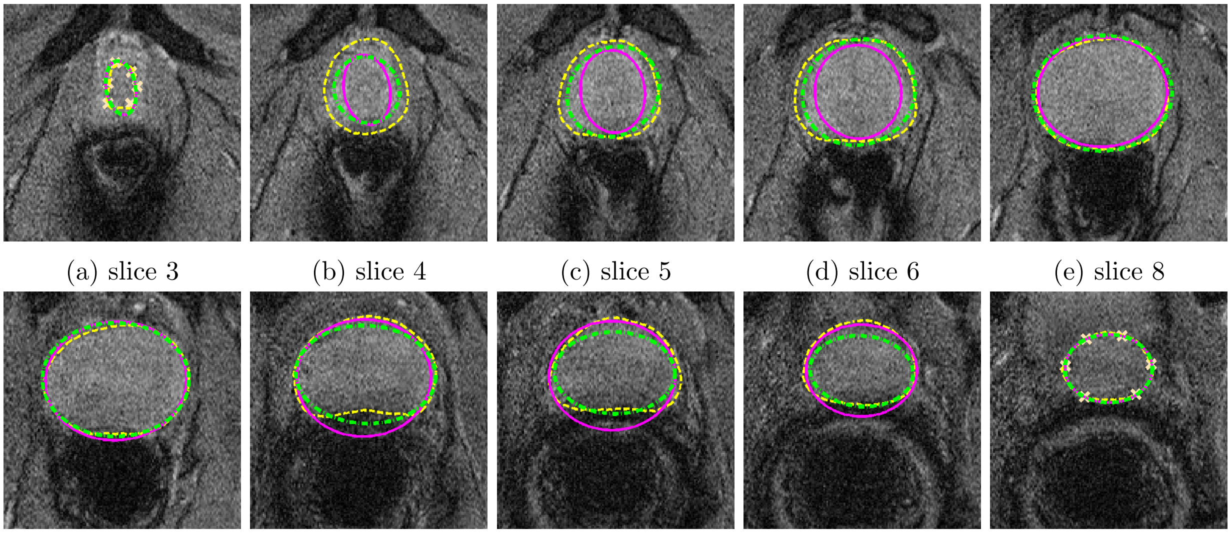

(a) slice 3 (b) slice 4 (c) slice 5 (d) slice 6 (e) slice 8

(f) slice 10 (g) slice 12 (h) slice 13 (i) slice 14 (j) slice 15

Figure 13: Selected MR slices of test data set 9 in Table 5 and 6. Slice 3 is the first slice and slice 15 the last slice with prostate information in the data set. The control point of the first and last slice are depicted in (a) and (j). Depicted are manual delineation line (yellow dashed line), deformed mean shape (magenta solid line) and result after MCMC (green dashed dotted line). Slices 7, 9 and 11 are not depitecd in the Figure.

The standard deviation of the error is chosen to be 50, i.e., $\epsilon \sim N(0, \sigma^2)$ with $\sigma = 50$.

In general, we can note the MCMC procedure works well in case of an ideal underlying image information. We do not need prior information if we use volume 2 to 5. The true underlying delineation can be found by the likelihood. Volume overlap and accuracy are close to the results obtained by the BFE for $\beta = 0$ which indicate the best possible values using the proposed model. Furthermore, we observe a decline of the volume overlap and accuracy for larger β using volume 2 to 5, and a convergence to the prior for $\beta \to \infty$. The convergence rate is different between both test data set which which can be explained by a less or more complex structure of the prostate. In addition, the convergence to the prior is faster using volume 2 and 3 compared to volume 4 and 5 due to the lower contrast in inside and outside of the object in volume 2 and 3. In case of the the original volume information (volume 1), the prior contains the most valuable information about the prostate contour. We can observe the largest improvement after MCMC for both data set in the range of $\beta = 20, 40$ and 60.

In Figure 14 is shown low information in the MR volumes is a limitation for the MCMC

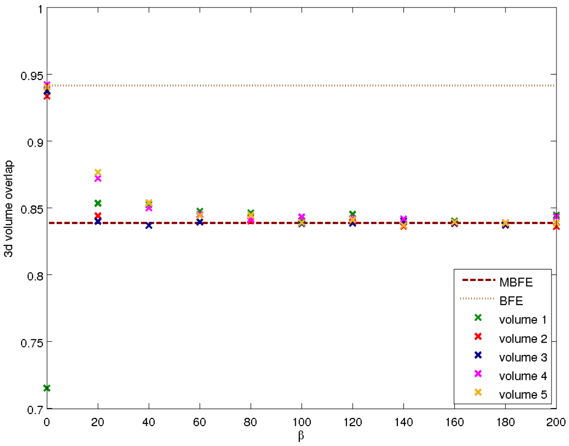

(a) test data set 6

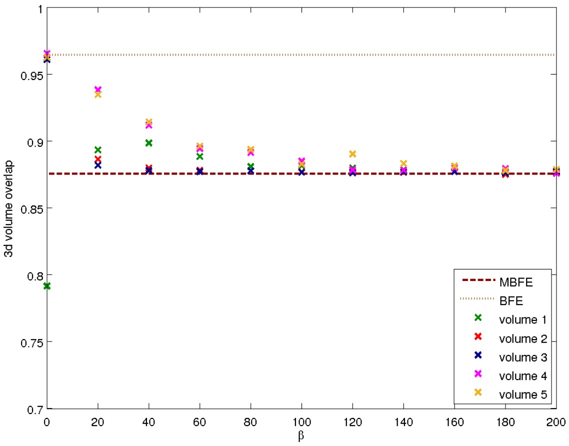

(b) test data set 8

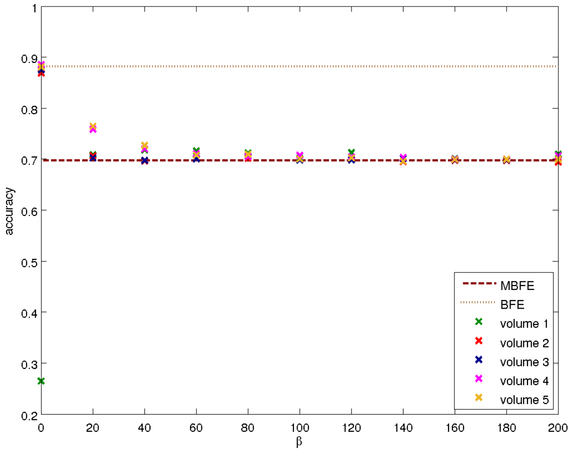

(c) test data set 6

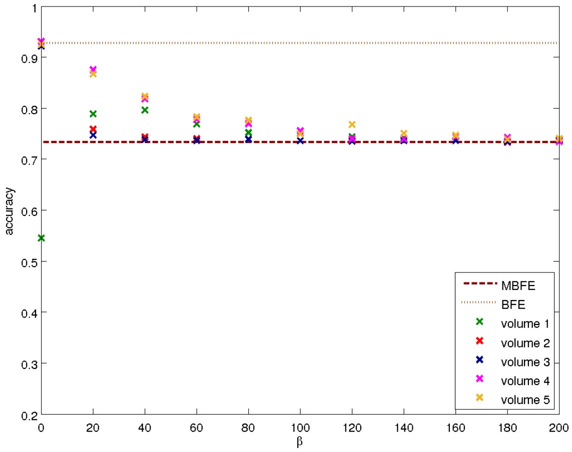

(d) test data set 8

Figure 14: Volume overlap ((a),(b)) and accuracy ((c),(d)) of the MCMC results with the manual delineation using different background images and for different β for test data sets 6 and 8. (volume 1): Using the original volume information. (volume 2): Derived volume by setting all gray values inside the best fitting ellipse to 100 and outside to 130 plus an error. (volume 3): Derived volume by setting all gray values inside the manual delineation line to 100 and outside to 130, plus an error. (volume 4): Derived volume by setting all gray values inside the best fitting ellipse to 80 and outside to 150, plus an error. (volume 5): Derived volume by setting all gray values inside the best fitting ellipse to 80 and outside to 150 plus an error.

approach. In addition, a careful choice of β, β_1, β_2 and β_3 is a difficult task. Several attempts to improve the MCMC results were carried out, e.g., different models for the likelihood, calculation of the Hastings ratio per slice instead of taking into account the entire volume and using the median instead of the mean to calculate the average of the gray values inside and outside the contour for the likelihood. Another idea was to select slices with a significant difference of the histogram inside

and outside of the deformed mean shape, e.g., by a Kolmogorv Smirnov test, a Andersson Darling test or by calculation of the earth mover's distance. All our attempts did not significantly improve the MCMC results for all test data sets so far.

Nevertheless, a potential improvement by a different likelihood model is a topic of further reaseach. Such a likelihood should model the underlying volume structure more carefully. Further possible directions are to use the MCMC method to sample from probability distributions on the space of curves as suggested by Fan et al. [5], to derive additional constraints from the training data set or to create an appearance model as suggested by Cootes and Taylor [6].

References

1. Altmann SL: *Rotations, quaternions, and double groups.* Dover books on mathematics, Dover Publications 2005.
2. Casella G: **An Introduction to Empirical Bayes Data Analsysis**. *The American Statistician* 1985, **39**(2):83–87.
3. Dryden IL, Mardia KV: *Statistical Shape Analysis.* John Wiley & Sons 1998.
4. Robert CP, Casella G: *Monte Carlo Statistical Method.* Springer, 2 edition 2004.
5. Fan AC, Fisher JWI, Kane J, Willsky AS: **MCMC curve sampling and geometric conditional simulationSPIE**. In *Computational Imaging VI*, *Volume 6814* 2008:681407–681407–12.
6. Cootes T, Taylor C: **Statistical Models of Appearance for Computer Vision**. Tech. rep., Imaging Science and Biomedical Engineering 2004, [http://www.isbe.man.ac.uk].
